# Supplementary material for: Conformational equilibria and intrinsic affinities define integrin activation
Source: EMBO J. 2017 Jan 25;36(5):629–45. doi: 10.15252/embj.201695803 (PMC5331762; doi:10.15252/embj.201695803)
Supplement: Supplementary file 1 — Appendix [file EMBJ-36-629-s001.pdf]

Conformational Equilibria and Intrinsic Affinities Define Integrin Activation

Jing Li[1*], Yang Su[1*], Wei Xia[1], Yan Qin[1], Martin Humphries[2], Dietmar Vestweber[3], Carlos Cabañas[4], Chafen Lu[1] and Timothy A. Springer[1]

[1]Program in Cellular and Molecular Medicine, Boston Children's Hospital and Department of Biological Chemistry and Molecular Pharmacology, Harvard Medical School, 3 Blackfan Circle, Boston, MA 02115

[2]Wellcome Trust Centre for Cell-Matrix Research, Faculty of Life Sciences, University of Manchester, M13 9PT, United Kingdom

[3]Max-Planck-Institute of Molecular Biomedicine, Germany

[4]Centro de Biología Molecular Severo Ochoa (CSIC-UAM), 28049 Madrid, Spain

Corresponding author:
Dr. Timothy A. Springer, Program in Cellular and Molecular Medicine at Boston Children's Hospital
Department of Biological Chemistry and Molecular Pharmacology, Harvard Medical School
3 Blackfan Circle, 3rd Floor, Room 3103, Boston, MA 02115
(617) 713-8200, Fax: (617) 713-8232
timothy.springer@childrens.harvard.edu
[*]Equal contribution

Contents

Materials and Methods . . . 2
- Materials . . . 2
- Negative stain electron microscopy (EM) . . . 2
- Isothermal titration calorimetry (ITC) . . . 2
- Protein and carbohydrate composition . . . 2
- Staining integrin subunits on cell surface . . . 2

Supplementary Text . . . 2
- EC_{50} or affinity of Fabs for $\alpha_5\beta_1$ and Eqs. S1–S11 . . . 2
- Affinity of $\alpha_5\beta_1$ for ligand or for 12G10 Fab from saturation binding and Eqs. S12–S17 . . . 4
- Affinity of intact $\alpha_5\beta_1$ for $Fn3_{9-10}$ and Eqs. S18–S26 . . . 5
- Affinity of $\alpha_5\beta_1$ ectodomain for $Fn3_{9-10}$ from competitive binding and Eqs. S27–S28 . . . 6
- Fab-binding affinities of $\alpha_5\beta_1$ conformational ensembles and Eqs. S29–S40 . . . 7
- True ligand-binding affinities (K_d^{ens}) of $\alpha_5\beta_1$ conformational ensemble members and Eqs. S41–S72 . . . 8
- Calculation of probability of each conformational state and Eqs. S73–S77 . . . 13
- Calculation of free energy of each conformational state and Eqs. S78–S84 . . . 14
- Calculation of free energies associated with conformational changes and Eqs. S85–S94 . . . 15

Supplemental References . . . 16

Supplemental Figures . . . 17

Materials and Methods

Materials

hTERT-BJ cells were a gift from Dr. Robert A. Weinberg (Whitehead Institute for Biomedical Research). Mouse anti-human antibodies J143 (4th International Workshop on Leukocytes), SAM-1 (5th International Workshop on Leukocytes), #481709 (R&D Systems), LM142 (EMD Millipore) and TS2/4 [1] were from the indicated sources. FITC-conjugated goat anti-mouse IgG was purchased from Sigma. α_5 specific blocking antibody mAb16 was purified from hybridoma provided to us by Dr. Kenneth M. Yamada. α_4 specific antibody Natalizumab was from commercial source.

Negative stain electron microscopy (EM)

EM specimen preparation, data collection and processing were as described [2].

Isothermal titration calorimetry (ITC)

ITC was performed in buffer containing 20 mM Tris (pH 7.4), 150 mM NaCl, 1 mM $CaCl_2$ and 1 mM $MgCl_2$ at 25 °C with 20 injections (2 µL each) on MicroCal iTC200. Data were fit to the one-site binding model [3] in OriginPro 7.

Protein and carbohydrate composition

Unclasped $\alpha_5\beta_1$ ectodomain with shaved (30 µg), high-mannose (60 µg), and complex (30 µg) N-glycans were separately loaded on an Agilent liquid chromatography system equipped with a TSKgel BioAssist G4SWXL analytical size exclusion column (Tosoh Bioscience), a DAWN HELEOS II multi-angle light scattering detector, an Optilab T-rEX refractive index detector and a variable wavelength UV detector (Wyatt Technology Corporation). Data were processed in ASTRA 6 using the protein conjugate model ($dn/dc = 0.185$ and 0.145 for protein and carbohydrate components, respectively) [4].

Staining integrin subunits on cell surface

Surface expression of integrin α_3-, α_5-, α_8- and α_V-subunits on K562, HEK293 and hTERT-BJ cells was quantified by immuno-staining. β_1 integrins with the latter three α-subunits bind ligands containing the Arg-Gly-Asp (RGD) motif [5]. Cells (10^6/mL in PBS supplemented with 50 mg/mL BSA and 1 mg/mL Na_3N) were incubated on ice with 50 µg/mL human IgG for 20 min to block Fc-receptors, then incubated with 2.5 µg/mL primary antibodies J143 (anti-α_3), SAM-1 (anti-α_5), #481709 (anti-α_8), LM142 (anti-α_V) or TS2/4 (anti-α_L) for 30 min, followed by 3 washes. Cells were then incubated with 2 µg/mL FITC-conjugated goat anti-mouse IgG for 30 min, followed by 3 washes, and subjected to flow cytometry (BD FACSCanto II). Staining lymphocyte integrin subunit α_L was a negative control.

Quantitative comparison of α_5-, α_4- and β_1 subunit expression levels on K562 and Jurkat cells was the same as described about except that cells were only incubated with 3.75 µg/mL Alexa647-conjugated primary antibodies before subject to washing and flow cytometry.

Supplementary Text

EC_{50} or affinity of Fabs for $\alpha_5\beta_1$ and Eqs. S1–S11

For soluble $\alpha_5\beta_1$ ectodomain or headpiece proteins, 20 nM $\alpha_5\beta_1$ (or 100 nM $\alpha_5\beta_1$ with closure-stabilizing Fabs) were equilibrated with 0–10,000 nM of Fabs for 2 hr. The mixture was then incubated with 5 nM

FITC-cRGD for 2 hr, and FP was measured. Fab-binding was reported by changes in FP. For intact $\alpha_5\beta_1$, K562 cells (2×10^6 cells/mL) were incubated with 10 nM Alexa488-Fn_{9-10} and 0–100,000 nM of Fabs for 1.5 hr and subjecting to flow cytometry. Fab-binding was reported by changes in mean fluorescence intensity (MFI).

For determining EC_{50} values for extension-stabilizing and open-stabilizing Fabs, we made the assumption that the increase in FP was directly proportional to the increase in concentration of Fab-bound open $\alpha_5\beta_1$. This assumption is reasonable because the affinity of the EO conformation is so much higher than that of the BC and EC conformations for cRGD. Therefore, data were fit to a dose response curve:

$$FP_{\mathrm{obs}} = FP_0 + \frac{FP_{\mathrm{sat}} - FP_0}{EC_{50}/[\mathrm{Fab}]_{\mathrm{tot}} + 1} \tag{S1}$$

$$MFI_{\mathrm{obs}} = \frac{MFI_{\mathrm{sat}}}{EC_{50}/[\mathrm{Fab}]_{\mathrm{tot}} + 1} \tag{S2}$$

where FP_0 is the FP without added Fab, FP_{sat} and MFI_{sat} are plateau values of FP and MFI, respectively, at high Fab concentration, and EC_{50} is the Fab concentration at the inflection point where half-maximum change in FP or MFI was observed. The Fab's K_{d} for $\alpha_5\beta_1$ is approximated by EC_{50}.

In the case of closure-stabilizing Fabs where $\alpha_5\beta_1$ was used at a high concentration in the assay, EC_{50} significantly deviates from K_{d} due to depletion of Fab and FITC-cRGD. Therefore, we wrote equations S3-S10 as described below, and fit data to Eq. S11 below. In the assay, FITC-cRGD and its complex with $\alpha_5\beta_1$ free of Fab were the major sources of FP_{obs}; the $\alpha_5\beta_1{\cdot}$Fab complex essentially does not bind FITC-cRGD due to its extremely low affinity, which was evident in Fig. 2 and Fig. S1 where at high concentrations of closure-stabilizing Fab, FP_{obs} dropped to the value of free FITC-cRGD (0.09). Experimentally, $\alpha_5\beta_1$ was at 100 nM and cRGD was at 5 nM; in experiments to determine Fab K_{d} (Fig. S1) most of the FP signal was due to cRGD bound to the open $\alpha_5\beta_1$ conformation. Because the observed decrease in FP was due to Fab-binding to $\alpha_5\beta_1$ and stabilizing it in the closed conformation, we first wrote the equations for Fab-binding to $\alpha_5\beta_1$ and then considered the effect on $\alpha_5\beta_1$ binding to cRGD:

$$\alpha_5\beta_1 + \mathrm{Fab} \rightleftharpoons \alpha_5\beta_1{\cdot}\mathrm{Fab} \qquad K_{\mathrm{d}}^{\mathrm{ens(Basal):Fab}} = \frac{[\alpha_5\beta_1]'[\mathrm{Fab}]}{[\alpha_5\beta_1{\cdot}\mathrm{Fab}]} \tag{S3}$$

$$[\alpha_5\beta_1]_{\mathrm{tot}} = [\alpha_5\beta_1]' + [\alpha_5\beta_1{\cdot}\mathrm{Fab}] \tag{S4}$$

$$[\mathrm{Fab}]_{\mathrm{tot}} = [\mathrm{Fab}] + [\alpha_5\beta_1{\cdot}\mathrm{Fab}] \tag{S5}$$

where $K_{\mathrm{d}}^{\mathrm{ens(Basal):Fab}}$ is Fab's K_{d} for $\alpha_5\beta_1$, $[\alpha_5\beta_1]_{\mathrm{tot}}$ and $[\mathrm{Fab}]_{\mathrm{tot}}$ are total concentrations of $\alpha_5\beta_1$ (100 nM) and closure-stabilizing Fab in the assay, respectively; $[\alpha_5\beta_1]'$ is the concentration of Fab-free $\alpha_5\beta_1$ at equilibrium. In the following equations, we make the reasonable assumption that only Fab-free $\alpha_5\beta_1$ contributes to the FP signal:

$$\alpha_5\beta_1 + \mathrm{L} \rightleftharpoons \alpha_5\beta_1{\cdot}\mathrm{L} \qquad K_{\mathrm{d}}^{\mathrm{ens(Basal):L}} = \frac{[\alpha_5\beta_1][\mathrm{L}]}{[\alpha_5\beta_1{\cdot}\mathrm{L}]} \tag{S6}$$

$$[\alpha_5\beta_1]' = [\alpha_5\beta_1] + [\alpha_5\beta_1{\cdot}\mathrm{L}] \tag{S7}$$

$$[\mathrm{L}]_{\mathrm{tot}} = [\mathrm{L}] + [\alpha_5\beta_1{\cdot}\mathrm{L}] \tag{S8}$$

where $[\mathrm{L}]_{\mathrm{tot}}$ is the total concentration of FITC-cRGD (5 nM); $[\alpha_5\beta_1]$ is the concentration of free $\alpha_5\beta_1$ in the final mixture, and $K_{\mathrm{d}}^{\mathrm{ens(Basal):L}}$ is the affinity of $\alpha_5\beta_1$ for FITC-cRGD in the absence of Fabs, which was measured separately in a saturation binding assay (next section).

Solve Eq. S3–S8 for $[\alpha_5\beta_1{\cdot}\mathrm{L}]$:

$$[\alpha_5\beta_1]' = \frac{[\alpha_5\beta_1]_{\mathrm{tot}} - [\mathrm{Fab}]_{\mathrm{tot}} - K_{\mathrm{d}}^{\mathrm{ens(Basal):Fab}} + \sqrt{\left([\alpha_5\beta_1]_{\mathrm{tot}} + [\mathrm{Fab}]_{\mathrm{tot}} + K_{\mathrm{d}}^{\mathrm{ens(Basal):Fab}}\right)^2 - 4[\alpha_5\beta_1]_{\mathrm{tot}}[\mathrm{Fab}]_{\mathrm{tot}}}}{2} \tag{S9}$$

$$[\alpha_5\beta_1{\cdot}\mathrm{L}] = \frac{[\alpha_5\beta_1]' + [\mathrm{L}]_{\mathrm{tot}} + K_{\mathrm{d}}^{\mathrm{ens(Basal):L}} - \sqrt{\left([\alpha_5\beta_1]' + [\mathrm{L}]_{\mathrm{tot}} + K_{\mathrm{d}}^{\mathrm{ens(Basal):L}}\right)^2 - 4[\alpha_5\beta_1]'[\mathrm{L}]_{\mathrm{tot}}}}{2} \tag{S10}$$

Therefore

$$FP_{\mathrm{obs}} = \frac{[\mathrm{L}]}{[\mathrm{L}]_{\mathrm{tot}}} \cdot FP_{\mathrm{L}} + \frac{[\alpha_5\beta_1{\cdot}\mathrm{L}]}{[\mathrm{L}]_{\mathrm{tot}}} \cdot FP_{\alpha_5\beta_1\cdot\mathrm{L}} \quad (^6)$$

$$= \frac{[\mathrm{L}]_{\mathrm{tot}} - [\alpha_5\beta_1{\cdot}\mathrm{L}]}{[\mathrm{L}]_{\mathrm{tot}}} \cdot FP_{\mathrm{L}} + \frac{[\alpha_5\beta_1{\cdot}\mathrm{L}]}{[\mathrm{L}]_{\mathrm{tot}}} \cdot FP_{\alpha_5\beta_1\cdot\mathrm{L}} = FP_{\mathrm{L}} + \frac{[\alpha_5\beta_1{\cdot}\mathrm{L}]}{[\mathrm{L}]_{\mathrm{tot}}} \cdot \left(FP_{\alpha_5\beta_1\cdot\mathrm{L}} - FP_{\mathrm{L}}\right)$$

$$= FP_{\mathrm{L}} + \frac{[\alpha_5\beta_1]' + [\mathrm{L}]_{\mathrm{tot}} + K_{\mathrm{d}}^{\mathrm{ens(Basal):L}} - \sqrt{\left([\alpha_5\beta_1]' + [\mathrm{L}]_{\mathrm{tot}} + K_{\mathrm{d}}^{\mathrm{ens(Basal):L}}\right)^2 - 4[\alpha_5\beta_1]'[\mathrm{L}]_{\mathrm{tot}}}}{2[\mathrm{L}]_{\mathrm{tot}}} \cdot \left(FP_{\alpha_5\beta_1\cdot\mathrm{L}} - FP_{\mathrm{L}}\right) \tag{S11}$$

where FP_{L} and $FP_{\alpha_5\beta_1\cdot\mathrm{L}}$ are FP of free FITC-cRGD and $\alpha_5\beta_1$·FITC-cRGD complex, respectively; $[\alpha_5\beta_1]'$ is defined in Eq. S9. Fitting the FP_{obs} and $[\mathrm{Fab}]_{\mathrm{tot}}$ data to Eq. S11 yielded $K_{\mathrm{d}}^{\mathrm{ens(Basal):Fab}}$, FP_{L} and $FP_{\alpha_5\beta_1\cdot\mathrm{L}}$.

<u>Affinity of $\alpha_5\beta_1$ for ligand or for 12G10 Fab from saturation binding and Eqs. S12–S17</u>

For soluble $\alpha_5\beta_1$ ectodomain or headpiece proteins, 0.1–10,000 nM $\alpha_5\beta_1$ were incubated with 5 nM FITC-cRGD or FITC-RGD ligand for 2 hr (24 hr in the presence of 12G10 Fab to reach equilibirum). Binding of FITC-cRGD or FITC-RGD was measured as FP. For intact $\alpha_5\beta_1$, K562 cells (2×10^6 cells/mL) were incubated with 0.1–100 nM Alexa488-Fn_{9-10} or Alexa488-12G10 Fab for 1.5 hr and subjected to flow cytometry. Binding of Alexa488-Fn_{9-10} or Alexa488-12G10 was measured as mean fluorescence intensity (MFI). Using L to denote the fluorescent ligand or 12G10 Fab, at equilibrium:

$$\alpha_5\beta_1 + \mathrm{L} \rightleftharpoons \alpha_5\beta_1{\cdot}\mathrm{L} \qquad K_{\mathrm{d}}^{\mathrm{app}} = \frac{[\alpha_5\beta_1][\mathrm{L}]}{[\alpha_5\beta_1{\cdot}\mathrm{L}]} \tag{S12}$$

$$[\alpha_5\beta_1]_{\mathrm{tot}} = [\alpha_5\beta_1] + [\alpha_5\beta_1{\cdot}\mathrm{L}] \tag{S13}$$

$$[\mathrm{L}]_{\mathrm{tot}} = [\mathrm{L}] + [\alpha_5\beta_1{\cdot}\mathrm{L}] \tag{S14}$$

where $[\alpha_5\beta_1]_{\mathrm{tot}}$ is the total concentration of soluble $\alpha_5\beta_1$ in solution or total amount of $\alpha_5\beta_1$ on cell surface, $[\mathrm{L}]_{\mathrm{tot}}$ is the total concentration of the fluorescent ligand or 12G10 Fab, and $K_{\mathrm{d}}^{\mathrm{app}}$ is the apparent binding affinity of $\alpha_5\beta_1$ for ligand or 12G10 (defined in Eq. S47).

Solve Eq. S12–S14 for $[\alpha_5\beta_1{\cdot}L]$:

$$[\alpha_5\beta_1{\cdot}L] = \frac{[L]_{tot} + K_d^{app} + [\alpha_5\beta_1]_{tot} - \sqrt{\left([L]_{tot} + K_d^{app} + [\alpha_5\beta_1]_{tot}\right)^2 - 4[L]_{tot}[\alpha_5\beta_1]_{tot}}}{2} \tag{S15}$$

Therefore, the measured FP (FP_{obs}) or MFI (MFI_{obs}) are

$$FP_{obs} = \frac{[L]}{[L]_{tot}} \cdot FP_L + \frac{[\alpha_5\beta_1{\cdot}L]}{[L]_{tot}} \cdot FP_{\alpha_5\beta_1{\cdot}L} \quad (^6)$$

$$= \frac{[L]_{tot} - [\alpha_5\beta_1{\cdot}L]}{[L]_{tot}} \cdot FP_L + \frac{[\alpha_5\beta_1{\cdot}L]}{[L]_{tot}} \cdot FP_{\alpha_5\beta_1{\cdot}L} = FP_L + \frac{[\alpha_5\beta_1{\cdot}L]}{[L]_{tot}} \cdot \left(FP_{\alpha_5\beta_1{\cdot}L} - FP_L\right)$$

$$= FP_L + \frac{[L]_{tot} + K_d^{app} + [\alpha_5\beta_1]_{tot} - \sqrt{\left([L]_{tot} + K_d^{app} + [\alpha_5\beta_1]_{tot}\right)^2 - 4[L]_{tot}[\alpha_5\beta_1]_{tot}}}{2[L]_{tot}} \cdot \left(FP_{\alpha_5\beta_1{\cdot}L} - FP_L\right) \tag{S16}$$

$$MFI_{obs} = \frac{[\alpha_5\beta_1{\cdot}L]}{[\alpha_5\beta_1]_{tot}} \cdot MFI_{max}$$

$$= \frac{[L]_{tot} + K_d^{app} + [\alpha_5\beta_1]_{tot} - \sqrt{\left([L]_{tot} + K_d^{app} + [\alpha_5\beta_1]_{tot}\right)^2 - 4[L]_{tot}[\alpha_5\beta_1]_{tot}}}{2[\alpha_5\beta_1]_{tot}} \cdot MFI_{max} \tag{S17}$$

where FP_L and $FP_{\alpha_5\beta_1{\cdot}L}$ are FP of free and $\alpha_5\beta_1$-bound FITC-cRGD or FITC-RGD, respectively, and MFI_{max} is the MFI when all $\alpha_5\beta_1$ on cell surface are bound with Alexa488-$Fn3_{9-10}$ or Alexa488-12G10.

Fitting the FP_{obs} and $[\alpha_5\beta_1]_{tot}$ data to Eq. S16 yielded K_d^{app}, FP_L and $FP_{\alpha_5\beta_1{\cdot}L}$. Fitting the MFI_{obs} and $[L]_{tot}$ data to Eq. S17 yielded K_d^{app} and MFI_{max}.

Affinity of intact $\alpha_5\beta_1$ for $Fn3_{9-10}$ and Eqs. S18–S26

K562 cells ($2{\times}10^6$ cells/mL) were equilibrated with 1–10,000 nM $Fn3_{9-10}$ for 1.5 hr, followed by incubation with 0.4 nM Alexa488-12G10 Fab for 1.5 hr, and were subjected to flow cytometry. Both 12G10 and $Fn3_{9-10}$ stabilize the extended-open conformation. Alexa488-12G10 was used at a low concentration of 0.4 nM such that it did not show detectable binding to K562 cells in the absence of $Fn3_{9-10}$ and could be used to report stabilization by $Fn3_{9-10}$ of $\alpha_5\beta_1$ in the open conformation, which was only detectable at $Fn3_{9-10}$ concentrations above 100 nM. Therefore, we described reporting of the $Fn3_{9-10}$-stabilized conformation of $\alpha_5\beta_1$ by changes in mean fluorescence intensity (MFI) of Alexa488-12G10 using the following equations in which $Fn3_{9-10}$ is denoted as L and Alexa488-12G10 is denoted as Fab:

$$\alpha_5\beta_1 + L \rightleftharpoons \alpha_5\beta_1{\cdot}L \qquad K_d^{ens(Basal):L} = \frac{[\alpha_5\beta_1]'[L]}{[\alpha_5\beta_1{\cdot}L]} \tag{S18}$$

$$[\alpha_5\beta_1]_{tot} = [\alpha_5\beta_1] + [\alpha_5\beta_1{\cdot}L]' \tag{S19}$$

$$[L]_{tot} = [L] + [\alpha_5\beta_1{\cdot}L]' \tag{S20}$$

where $[\alpha_5\beta_1]_{tot}$ is the total amount of $\alpha_5\beta_1$ on cell surface, $[L]_{tot}$ is the total concentration of $Fn3_{9-10}$ in the assay, $[\alpha_5\beta_1{\cdot}L]'$ is the concentration of $\alpha_5\beta_1{\cdot}Fn3_{9-10}$ complex at equilibrium, and $K_d^{ens(Basal):L}$ is the affinity

for $Fn3_{9-10}$.

$$\alpha_5\beta_1{\cdot}L + Fab \rightleftharpoons \alpha_5\beta_1{\cdot}L{\cdot}Fab \qquad K_d^{EO:Fab} = \frac{[\alpha_5\beta_1{\cdot}L][Fab]}{[\alpha_5\beta_1{\cdot}L{\cdot}Fab]} \tag{S21}$$

$$[\alpha_5\beta_1{\cdot}L]' = [\alpha_5\beta_1{\cdot}L] + [\alpha_5\beta_1{\cdot}L{\cdot}Fab] \tag{S22}$$

$$[Fab]_{tot} = [Fab] + [\alpha_5\beta_1{\cdot}L{\cdot}Fab] \tag{S23}$$

where $[Fab]_{tot}$ is the total concentration of Alexa488-12G10 (0.4 nM); $[\alpha_5\beta_1{\cdot}L]$ is the final concentration of Alexa488-12G10-free $\alpha_5\beta_1{\cdot}Fn3_{9-10}$ complex in the final mixture, and $K_d^{EO:Fab}$ is the affinity of $\alpha_5\beta_1{\cdot}Fn3_{9-10}$ complex (in the extended-open conformation) for Alexa488-12G10, which was measured separately in a saturation binding assay (previous section).

Solve Eq. S18–S23 for $[\alpha_5\beta_1{\cdot}L{\cdot}Fab]$:

$$[\alpha_5\beta_1{\cdot}L]' = \frac{[\alpha_5\beta_1]_{tot} + [L]_{tot} + K_d^{ens(Basal):L} - \sqrt{\left([\alpha_5\beta_1]_{tot} + [L]_{tot} + K_d^{ens(Basal):L}\right)^2 - 4[\alpha_5\beta_1]_{tot}[L]_{tot}}}{2} \tag{S24}$$

$$[\alpha_5\beta_1{\cdot}L{\cdot}Fab] = \frac{[\alpha_5\beta_1{\cdot}L]' + [Fab]_{tot} + K_d^{EO:Fab} - \sqrt{\left([\alpha_5\beta_1{\cdot}L]' + [Fab]_{tot} + K_d^{EO:Fab}\right)^2 - 4[\alpha_5\beta_1{\cdot}L]'[Fab]_{tot}}}{2} \tag{S25}$$

Therefore, the measured MFI (MFI_{obs}) is

$$\begin{aligned} MFI_{obs} &= \frac{[\alpha_5\beta_1{\cdot}L{\cdot}Fab]}{[\alpha_5\beta_1]_{tot}} \cdot MFI_{max} \\ &= \frac{[\alpha_5\beta_1{\cdot}L]' + [Fab]_{tot} + K_d^{EO:Fab} - \sqrt{\left([\alpha_5\beta_1{\cdot}L]' + [Fab]_{tot} + K_d^{EO:Fab}\right)^2 - 4[\alpha_5\beta_1{\cdot}L]'[Fab]_{tot}}}{2[\alpha_5\beta_1]_{tot}} \cdot MFI_{max} \end{aligned} \tag{S26}$$

where MFI_{max} is the MFI when all $\alpha_5\beta_1$ on cell surface is bound to Alexa488-12G10.

Fitting the MFI_{obs} and $[L]_{tot}$ to Eq. S26 yielded $K_d^{ens(Basal):L}$ and MFI_{max}.

Affinity of $\alpha_5\beta_1$ ectodomain for $Fn3_{9-10}$ from competitive binding and Eqs. S27–S28

$\alpha_5\beta_1$ ectodomain affinities for $Fn3_{9-10}$ was measured by using $Fn3_{9-10}$ to compete binding of FITC-cRGD peptide ligand. $\alpha_5\beta_1$ ectodomain (270 nM in the absence of Fabs, 20 nM in the presence of HUTS4 Fab, 90 nM in the presence of 8E3 Fab, or 70–10,000 nM in the presence of mAb13 Fab or mAb13 plus 9EG7 Fabs) was equilibrated with 0–10,000 nM $Fn3_{9-10}$ (competitor) for 2 hr. The mixture was incubated with 5 nM FITC-cRGD (ligand) for 2 hr, and FP was measured.

Since only $\alpha_5\beta_1$ free of $Fn3_{9-10}$ could bind FITC-cRGD, the equations are identical to those for binding affinities for closure-stabilizing Fabs (Eq. S3–S11). Substituting Fab with C (for competitor) in Eq. S9 and S11:

$$[\alpha_5\beta_1]' = \frac{[\alpha_5\beta_1]_{tot} - [C]_{tot} - K_d^{app:C} + \sqrt{\left([\alpha_5\beta_1]_{tot} + [C]_{tot} + K_d^{app:C}\right)^2 - 4[\alpha_5\beta_1]_{tot}[C]_{tot}}}{2} \tag{S27}$$

$$FP_{obs} = FP_L + \frac{[\alpha_5\beta_1]' + [L]_{tot} + K_d^{app:L} - \sqrt{\left([\alpha_5\beta_1]' + [L]_{tot} + K_d^{app:L}\right)^2 - 4[\alpha_5\beta_1]'[L]_{tot}}}{2[L]_{tot}} \cdot \left(FP_{\alpha_5\beta_1\cdot L} - FP_L\right) \tag{S28}$$

where FP_{obs} is the measured FP; FP_L and $FP_{\alpha_5\beta_1 \cdot L}$ are FP of free FITC-cRGD and $\alpha_5\beta_1$·FITC-cRGD complex, respectively; $[\alpha_5\beta_1]_{tot}$, $[L]_{tot}$ and $[C]_{tot}$ are total concentrations of $\alpha_5\beta_1$, FITC-cRGD ligand (5 nM) and $Fn3_{9-10}$ competitor in the assay, respectively; $[\alpha_5\beta_1]'$ is the concentration of $Fn3_{9-10}$-free $\alpha_5\beta_1$, either free of FITC-cRGD or with FITC-cRGD bound; $K_d^{app:C}$ and $K_d^{app:L}$ are apparent affinities of $\alpha_5\beta_1$ for $Fn3_{9-10}$ competitor and FITC-cRGD ligand, respectively.

For experiments in the absence of Fabs, or in the presence of HUTS4 or 8E3 Fab, fitting the FP_{obs} and $[C]_{tot}$ data to Eq. S28 using the $K_d^{app:L}$ value measured separately in a saturation binding assay yielded $K_d^{app:C}$, FP_L and $FP_{\alpha_5\beta_1 \cdot L}$. For experiments in the presence of mAb13 Fab or mAb13 plus 9EG7 Fabs, global fitting of the FP_{obs} and $[\alpha_5\beta_1]_{tot}$ data at different fixed $[C]_{tot}$ to Eq. S28 yielded $K_d^{app:C}$, $K_d^{app:L}$, FP_L and $FP_{\alpha_5\beta_1 \cdot L}$

Fab-binding affinities of $\alpha_5\beta_1$ conformational ensembles and Eqs. S29–S40

The basal ensemble (i.e., in the absence of Fabs) of intact $\alpha_5\beta_1$ on the cell surface or of its ectodomain fragment in solution comprises three overall conformational states—bent-closed (BC), extended-closed (EC), and extended-open (EO). Suppose a Fab binds (and thus stabilizes) one or more of the BC, EC and EO states in the basal ensemble to form BC·Fab, EC·Fab and/or EO·Fab complexes with intrinsic Fab-binding affinities K_a^{BC}, K_a^{EC} and/or K_a^{EO}, respectively:

$$\mathrm{BC} + \mathrm{Fab} \rightleftharpoons \mathrm{BC{\cdot}Fab} \qquad K_a^{BC} = \frac{[\mathrm{BC{\cdot}Fab}]}{[\mathrm{BC}][\mathrm{Fab}]} \tag{S29}$$

$$\mathrm{EC} + \mathrm{Fab} \rightleftharpoons \mathrm{EC{\cdot}Fab} \qquad K_a^{EC} = \frac{[\mathrm{EC{\cdot}Fab}]}{[\mathrm{EC}][\mathrm{Fab}]} \tag{S30}$$

$$\mathrm{EO} + \mathrm{Fab} \rightleftharpoons \mathrm{EO{\cdot}Fab} \qquad K_a^{EO} = \frac{[\mathrm{EO{\cdot}Fab}]}{[\mathrm{EO}][\mathrm{Fab}]} \tag{S31}$$

If the Fab does not bind a state i (i = BC, EC or EO), then $K_a^i = 0$ and $[i{\cdot}\mathrm{Fab}] = 0$. For Fabs that bind two states i and j with equal affinities, i.e., $K_a^i = K_a^j$ (i = EC and j = EO for extension-stabilizing Fabs, i = BC and j = EC for closure-stabilizing Fabs), Fab-binding does not change the relative distribution of the two states:

$$\frac{[i{\cdot}\mathrm{Fab}]}{[j{\cdot}\mathrm{Fab}]} = \frac{K_a^i[i][\mathrm{Fab}]}{K_a^j[j][\mathrm{Fab}]} = \frac{[i]}{[j]} \tag{S32}$$

The experimentally measured affinity of the basal ensemble for Fab, $K_a^{ens(Basal)}$, is a probability-weighted average of K_a^{BC}, K_a^{EC} and K_a^{EO}:

$$\begin{aligned} K_a^{ens(Basal)} &= \frac{[\mathrm{BC{\cdot}Fab}] + [\mathrm{EC{\cdot}Fab}] + [\mathrm{EO{\cdot}Fab}]}{([\mathrm{BC}] + [\mathrm{EC}] + [\mathrm{EO}])\,[\mathrm{Fab}]} \\ &= \frac{[\mathrm{BC}]}{[\mathrm{BC}] + [\mathrm{EC}] + [\mathrm{EO}]} \cdot \frac{[\mathrm{BC{\cdot}Fab}]}{[\mathrm{BC}][\mathrm{Fab}]} + \\ &\quad \frac{[\mathrm{EC}]}{[\mathrm{BC}] + [\mathrm{EC}] + [\mathrm{EO}]} \cdot \frac{[\mathrm{EC{\cdot}Fab}]}{[\mathrm{EC}][\mathrm{Fab}]} + \\ &\quad \frac{[\mathrm{EO}]}{[\mathrm{BC}] + [\mathrm{EC}] + [\mathrm{EO}]} \cdot \frac{[\mathrm{EO{\cdot}Fab}]}{[\mathrm{EO}][\mathrm{Fab}]} \\ &= P^{BC}K_a^{BC} + P^{EC}K_a^{EC} + P^{EO}K_a^{EO} \end{aligned} \tag{S33}$$

where P^{BC}, P^{EC} and P^{EO} are probabilities (populations) of the corresponding conformational states in the basal ensemble:

$$P^{\mathrm{BC}} = \frac{[\mathrm{BC}]}{[\mathrm{BC}] + [\mathrm{EC}] + [\mathrm{EO}]} \tag{S34}$$

$$P^{\mathrm{EC}} = \frac{[\mathrm{EC}]}{[\mathrm{BC}] + [\mathrm{EC}] + [\mathrm{EO}]} \tag{S35}$$

$$P^{\mathrm{EO}} = \frac{[\mathrm{EO}]}{[\mathrm{BC}] + [\mathrm{EC}] + [\mathrm{EO}]} \tag{S36}$$

$$P^{\mathrm{BC}} + P^{\mathrm{EC}} + P^{\mathrm{EO}} = 1 \tag{S37}$$

Because $K_{\mathrm{a}}^{\mathrm{BC}}$, $K_{\mathrm{a}}^{\mathrm{EC}}$, $K_{\mathrm{a}}^{\mathrm{EO}}$, P^{BC}, P^{EC} and P^{EO} are all constants of the equilibria in the ensemble, Eq. S33 shows that $K_{\mathrm{a}}^{\mathrm{ens(Basal)}}$ is a genuine equilibrium constant.

Binding affinity is often expressed as dissociation constant ($K_{\mathrm{d}} = 1/K_{\mathrm{a}}$) in the biological sciences to facilitate comparison to concentrations of the reactants. Following this convention, Eq. S33 can be rewritten as:

$$\frac{1}{K_{\mathrm{d}}^{\mathrm{ens(Basal)}}} = \frac{P^{\mathrm{BC}}}{K_{\mathrm{d}}^{\mathrm{BC}}} + \frac{P^{\mathrm{EC}}}{K_{\mathrm{d}}^{\mathrm{EC}}} + \frac{P^{\mathrm{EO}}}{K_{\mathrm{d}}^{\mathrm{EO}}} \tag{S38}$$

The total probability (population) of all Fab-bound species in the ensemble is

$$\begin{aligned} P^{\alpha_5\beta_1\cdot\mathrm{Fab}} &= \frac{[\mathrm{BC{\cdot}Fab}] + [\mathrm{EC{\cdot}Fab}] + [\mathrm{EO{\cdot}Fab}]}{[\mathrm{BC}] + [\mathrm{EC}] + [\mathrm{EO}] + [\mathrm{BC{\cdot}Fab}] + [\mathrm{EC{\cdot}Fab}] + [\mathrm{EO{\cdot}Fab}]} \\ &= \frac{1}{\dfrac{[\mathrm{BC}] + [\mathrm{EC}] + [\mathrm{EO}]}{[\mathrm{BC{\cdot}Fab}] + [\mathrm{EC{\cdot}Fab}] + [\mathrm{EO{\cdot}Fab}]} + 1} \\ &= \frac{1}{K_{\mathrm{d}}^{\mathrm{ens(Basal)}}/[\mathrm{Fab}] + 1} \end{aligned} \tag{S39}$$

Eq. S39 shows that if the Fab concentration is sufficiently high such that $K_{\mathrm{d}}^{\mathrm{ens(Basal)}}/[\mathrm{Fab}] \ll 1$, then $P^{\alpha_5\beta_1\cdot\mathrm{Fab}} \sim 1$ and the ensemble is dominated by the Fab-stabilized state(s).

For the $\alpha_5\beta_1$ headpiece, a similar analysis shows that at a sufficiently high concentration of Fab, the Fab-stabilized state dominates the ensemble.

In Eq. S39, [Fab] is the concentration of free Fab. In most experiments, Fabs are used at concentrations much higher than that of $\alpha_5\beta_1$; therefore, [Fab] can be approximated as the total Fab concentration minus the highest $\alpha_5\beta_1$ concentration used ($[\mathrm{Fab}]_{\mathrm{tot}} - [\alpha_5\beta_1]_{\mathrm{tot}}$) in calculating $P^{\alpha_5\beta_1\cdot\mathrm{Fab}}$:

$$P^{\alpha_5\beta_1\cdot\mathrm{Fab}} = \frac{1}{K_{\mathrm{d}}^{\mathrm{ens(Basal)}}/\left([\mathrm{Fab}]_{\mathrm{tot}} - [\alpha_5\beta_1]_{\mathrm{tot}}\right) + 1} \tag{S40}$$

<u>True ligand-binding affinities ($K_{\mathrm{d}}^{\mathrm{ens}}$) of $\alpha_5\beta_1$ conformational ensemble members and Eqs. S41–S72</u>

We now consider using Fabs to stabilize conformational ensembles in specific states. A complication is that in the absence of 100% binding of the Fab, unbound $\alpha_5\beta_1$ can exist in other states. We derive the equations for determining the contributions of both Fab-bound $\alpha_5\beta_1$ ($P^{\alpha_5\beta_1\cdot\mathrm{Fab}}$) and unbound $\alpha_5\beta_1$ ($1 - P^{\alpha_5\beta_1\cdot\mathrm{Fab}}$) to the measured apparent affinities ($K_{\mathrm{d}}^{\mathrm{app}}$). We show that under our experimental conditions, with open-stabilizing

and extension-stabilizing Fabs, $P^{\alpha_5\beta_1 \cdot \mathrm{Fab}}$ is 95–99.6%, and $K_\mathrm{d}^\mathrm{app} \simeq K_\mathrm{d}^\mathrm{ens}$ (Fig. S2). With closure-stabilizing Fabs under our experimental conditions, $P^{\alpha_5\beta_1 \cdot \mathrm{Fab}}$ is 99.8–99.9%, $K_\mathrm{d}^\mathrm{app}$ can differ from $K_\mathrm{d}^\mathrm{ens}$ (Fig. S2), and we use our derived equations to calculate true affinity, $K_\mathrm{d}^\mathrm{ens}$.

Intact $\alpha_5\beta_1$ or its ectodomain fragment contain three states in their conformational ensemble, BC, EC and EO. Suppose they bind a ligand, L, to form the BC·L, EC·L, and EO·L complexes with intrinsic ligand-binding affinity (association constants) K_a^BC, K_a^EC and K_a^EO, respectively:

$$\mathrm{BC} + \mathrm{L} \rightleftharpoons \mathrm{BC{\cdot}L} \qquad K_\mathrm{a}^\mathrm{BC} = \frac{[\mathrm{BC{\cdot}L}]}{[\mathrm{BC}][\mathrm{L}]} \tag{S41}$$

$$\mathrm{EC} + \mathrm{L} \rightleftharpoons \mathrm{EC{\cdot}L} \qquad K_\mathrm{a}^\mathrm{EC} = \frac{[\mathrm{EC{\cdot}L}]}{[\mathrm{EC}][\mathrm{L}]} \tag{S42}$$

$$\mathrm{EO} + \mathrm{L} \rightleftharpoons \mathrm{EO{\cdot}L} \qquad K_\mathrm{a}^\mathrm{EO} = \frac{[\mathrm{EO{\cdot}L}]}{[\mathrm{EO}][\mathrm{L}]} \tag{S43}$$

If a Fab is also present in the ensemble, then the Fab-bound state(s) BC·Fab, EC·Fab and/or EO·Fab can bind the ligand L to form BC·Fab·L, EC·Fab·L, and/or EO·Fab·L complexes with intrinsic ligand-binding affinity (association constants) $K_\mathrm{a}^\mathrm{BC\cdot Fab}$, $K_\mathrm{a}^\mathrm{EC\cdot Fab}$ and $K_\mathrm{a}^\mathrm{EO\cdot Fab}$, respectively. Suppose the Fab-bound $\alpha_5\beta_1$ states bind ligand with the same affinity as their corresponding native $\alpha_5\beta_1$ states (see main text for justification), then:

$$\mathrm{BC{\cdot}Fab} + \mathrm{L} \rightleftharpoons \mathrm{BC{\cdot}Fab{\cdot}L} \qquad K_\mathrm{a}^\mathrm{BC\cdot Fab} = \frac{[\mathrm{BC{\cdot}Fab{\cdot}L}]}{[\mathrm{BC{\cdot}Fab}][\mathrm{L}]} = K_\mathrm{a}^\mathrm{BC} \tag{S44}$$

$$\mathrm{EC{\cdot}Fab} + \mathrm{L} \rightleftharpoons \mathrm{EC{\cdot}Fab{\cdot}L} \qquad K_\mathrm{a}^\mathrm{EC\cdot Fab} = \frac{[\mathrm{EC{\cdot}Fab{\cdot}L}]}{[\mathrm{EC{\cdot}Fab}][\mathrm{L}]} = K_\mathrm{a}^\mathrm{EC} \tag{S45}$$

$$\mathrm{EO{\cdot}Fab} + \mathrm{L} \rightleftharpoons \mathrm{EO{\cdot}Fab{\cdot}L} \qquad K_\mathrm{a}^\mathrm{EO\cdot Fab} = \frac{[\mathrm{EO{\cdot}Fab{\cdot}L}]}{[\mathrm{EO{\cdot}Fab}][\mathrm{L}]} = K_\mathrm{a}^\mathrm{EO} \tag{S46}$$

If the Fab does not bind a state i (i = BC, EC or EO), then $[i{\cdot}\mathrm{Fab}] = 0$, $[i{\cdot}\mathrm{Fab{\cdot}L}] = 0$ and $K_\mathrm{a}^{i\cdot\mathrm{Fab}}$ is undefined.

Similarly to the earlier treatment of Fab-binding affinty (Eq. S33), it can be shown that the measured ligand-binding affinity of the ensemble, $K_\mathrm{a}^\mathrm{app}$, is ultimately a composite of intrinsic ligand-binding affinities:

$$\begin{aligned}
K_\mathrm{a}^\mathrm{app} &= \frac{[\mathrm{BC{\cdot}L}] + [\mathrm{EC{\cdot}L}] + [\mathrm{EO{\cdot}L}] + [\mathrm{BC{\cdot}Fab{\cdot}L}] + [\mathrm{EC{\cdot}Fab{\cdot}L}] + [\mathrm{EO{\cdot}Fab{\cdot}L}]}{([\mathrm{BC}] + [\mathrm{EC}] + [\mathrm{EO}] + [\mathrm{BC{\cdot}Fab}] + [\mathrm{EC{\cdot}Fab}] + [\mathrm{EO{\cdot}Fab}])\,[\mathrm{L}]} \\
&= \frac{[\mathrm{BC}] + [\mathrm{EC}] + [\mathrm{EO}]}{[\mathrm{BC}] + [\mathrm{EC}] + [\mathrm{EO}] + [\mathrm{BC{\cdot}Fab}] + [\mathrm{EC{\cdot}Fab}] + [\mathrm{EO{\cdot}Fab}]} \cdot \frac{[\mathrm{BC{\cdot}L}] + [\mathrm{EC{\cdot}L}] + [\mathrm{EO{\cdot}L}]}{([\mathrm{BC}] + [\mathrm{EC}] + [\mathrm{EO}])\,[\mathrm{L}]} + \\
&\quad \frac{[\mathrm{BC{\cdot}Fab}] + [\mathrm{EC{\cdot}Fab}] + [\mathrm{EO{\cdot}Fab}]}{[\mathrm{BC}] + [\mathrm{EC}] + [\mathrm{EO}] + [\mathrm{BC{\cdot}Fab}] + [\mathrm{EC{\cdot}Fab}] + [\mathrm{EO{\cdot}Fab}]} \cdot \frac{[\mathrm{BC{\cdot}Fab{\cdot}L}] + [\mathrm{EC{\cdot}Fab{\cdot}L}] + [\mathrm{EO{\cdot}Fab{\cdot}L}]}{([\mathrm{BC{\cdot}Fab}] + [\mathrm{EC{\cdot}Fab}] + [\mathrm{EO{\cdot}Fab}])\,[\mathrm{L}]} \\
&= \left(1 - \frac{[\mathrm{BC{\cdot}Fab}] + [\mathrm{EC{\cdot}Fab}] + [\mathrm{EO{\cdot}Fab}]}{[\mathrm{BC}] + [\mathrm{EC}] + [\mathrm{EO}] + [\mathrm{BC{\cdot}Fab}] + [\mathrm{EC{\cdot}Fab}] + [\mathrm{EO{\cdot}Fab}]}\right) \cdot \frac{[\mathrm{BC{\cdot}L}] + [\mathrm{EC{\cdot}L}] + [\mathrm{EO{\cdot}L}]}{([\mathrm{BC}] + [\mathrm{EC}] + [\mathrm{EO}])\,[\mathrm{L}]} + \\
&\quad \frac{[\mathrm{BC{\cdot}Fab}] + [\mathrm{EC{\cdot}Fab}] + [\mathrm{EO{\cdot}Fab}]}{[\mathrm{BC}] + [\mathrm{EC}] + [\mathrm{EO}] + [\mathrm{BC{\cdot}Fab}] + [\mathrm{EC{\cdot}Fab}] + [\mathrm{EO{\cdot}Fab}]} \cdot \frac{[\mathrm{BC{\cdot}Fab{\cdot}L}] + [\mathrm{EC{\cdot}Fab{\cdot}L}] + [\mathrm{EO{\cdot}Fab{\cdot}L}]}{([\mathrm{BC{\cdot}Fab}] + [\mathrm{EC{\cdot}Fab}] + [\mathrm{EO{\cdot}Fab}])\,[\mathrm{L}]} \\
&= \left(1 - P^{\alpha_5\beta_1\cdot\mathrm{Fab}}\right) \cdot K_\mathrm{a}^\mathrm{ens(Basal)} + P^{\alpha_5\beta_1\cdot\mathrm{Fab}} \cdot K_\mathrm{a}^\mathrm{ens(Fab\text{-}stabilized\ states)}
\end{aligned} \tag{S47}$$

where $P^{\alpha_5\beta_1\cdot\text{Fab}}$ is defined in Eq. S39, $K_a^{\text{ens(Basal)}}$ and $K_a^{\text{ens(Fab-stabilized states)}}$, as follows, are ligand-binding affinities of the basal ensemble and the ensemble of all Fab-stabilized state(s), respectively:

$$
\begin{aligned}
K_a^{\text{ens(Basal)}} &= \frac{[\text{BC·L}] + [\text{EC·L}] + [\text{EO·L}]}{([\text{BC}] + [\text{EC}] + [\text{EO}])\,[\text{L}]} \\
&= \frac{[\text{BC}]}{[\text{BC}] + [\text{EC}] + [\text{EO}]} \cdot \frac{[\text{BC·L}]}{[\text{BC}][\text{L}]} + \\
&\quad \frac{[\text{EC}]}{[\text{BC}] + [\text{EC}] + [\text{EO}]} \cdot \frac{[\text{EC·L}]}{[\text{EC}][\text{L}]} + \\
&\quad \frac{[\text{EO}]}{[\text{BC}] + [\text{EC}] + [\text{EO}]} \cdot \frac{[\text{EO·L}]}{[\text{EO}][\text{L}]} \\
&= P^{\text{BC}} K_a^{\text{BC}} + P^{\text{EC}} K_a^{\text{EC}} + P^{\text{EO}} K_a^{\text{EO}}
\end{aligned} \tag{S48}
$$

$$
\begin{aligned}
K_a^{\text{ens(Fab-stabilized states)}} &= \frac{[\text{BC·Fab·L}] + [\text{EC·Fab·L}] + [\text{EO·Fab·L}]}{([\text{BC·Fab}] + [\text{EC·Fab}] + [\text{EO·Fab}])\,[\text{L}]} \\
&= \frac{[\text{BC·Fab}]}{[\text{BC·Fab}] + [\text{EC·Fab}] + [\text{EO·Fab}]} \cdot \frac{[\text{BC·Fab·L}]}{[\text{BC·Fab}][\text{L}]} + \\
&\quad \frac{[\text{EC·Fab}]}{[\text{BC·Fab}] + [\text{EC·Fab}] + [\text{EO·Fab}]} \cdot \frac{[\text{EC·Fab·L}]}{[\text{EC·Fab}][\text{L}]} + \\
&\quad \frac{[\text{EO·Fab}]}{[\text{BC·Fab}] + [\text{EC·Fab}] + [\text{EO·Fab}]} \cdot \frac{[\text{EO·Fab·L}]}{[\text{EO·Fab}][\text{L}]} \\
&= P^{\text{BC·Fab}} K_a^{\text{BC}} + P^{\text{EC·Fab}} K_a^{\text{EC}} + P^{\text{EO·Fab}} K_a^{\text{EO}}
\end{aligned} \tag{S49}
$$

where the probabilities P^{BC}, P^{EC} and P^{EO} are defined in Eq. S34–S36, the intrinsic ligand-binding affinities K_a^{BC}, K_a^{EC} and K_a^{EO} are defined in Eq. S41–S43, and the probabilities $P^{\text{BC·Fab}}$, $P^{\text{EC·Fab}}$ and $P^{\text{EO·Fab}}$ are as follows:

$$
P^{\text{BC·Fab}} = \frac{[\text{BC·Fab}]}{[\text{BC·Fab}] + [\text{EC·Fab}] + [\text{EO·Fab}]} \tag{S50}
$$

$$
P^{\text{EC·Fab}} = \frac{[\text{EC·Fab}]}{[\text{BC·Fab}] + [\text{EC·Fab}] + [\text{EO·Fab}]} \tag{S51}
$$

$$
P^{\text{EO·Fab}} = \frac{[\text{EO·Fab}]}{[\text{BC·Fab}] + [\text{EC·Fab}] + [\text{EO·Fab}]} \tag{S52}
$$

$$
P^{\text{BC·Fab}} + P^{\text{EC·Fab}} + P^{\text{EO·Fab}} = 1 \tag{S53}
$$

Eq. S47 can be rewritten using dissociation constants:

$$
\frac{1}{K_d^{\text{app}}} = \frac{1 - P^{\alpha_5\beta_1\cdot\text{Fab}}}{K_d^{\text{ens(Basal)}}} + \frac{P^{\alpha_5\beta_1\cdot\text{Fab}}}{K_d^{\text{ens(Fab-stabilized states)}}} \tag{S54}
$$

In the absence of Fabs (i.e., $P^{\alpha_5\beta_1\cdot\text{Fab}} = 0$), Eq. S54 degenerates to $K_d^{\text{app}} = K_d^{\text{ens(Basal)}}$, which confirms the obvious fact that, in the absence of Fabs, the basal ensemble's ligand-binding affinity $K_d^{\text{ens(Basal)}}$ is

determined experimentally. By separately accounting for the contributions to K_d^{app} of $\alpha_5\beta_1$ both bound to Fab ($P^{\alpha_5\beta_1 \cdot Fab}$) and not bound to Fab ($1 - P^{\alpha_5\beta_1 \cdot Fab}$), we may calculate true affinities of Fab-stabilized individual states (K_d^{EC} and K_d^{EO}) or ensembles of two states ($K_d^{ens(BC+EC)}$ and $K_d^{ens(EC+EO)}$). In other words, K_d^{app} determined in the presence of Fabs, together with $K_d^{ens(Basal)}$ and $P^{\alpha_5\beta_1 \cdot Fab}$ (see previous section and Table S1), can be used to calculate $K_d^{ens(Fab\text{-}stabilized\ states)}$ by solving Eq. S54:

$$K_d^{ens(Fab\text{-}stabilized\ states)} = \frac{P^{\alpha_5\beta_1 \cdot Fab} K_d^{ens(Basal)} K_d^{app}}{K_d^{ens(Basal)} - \left(1 - P^{\alpha_5\beta_1 \cdot Fab}\right) K_d^{app}} \tag{S55}$$

Eq. S48–S49 can be rewritten using dissociation constants:

$$\frac{1}{K_d^{ens(Basal)}} = \frac{P^{BC}}{K_d^{BC}} + \frac{P^{EC}}{K_d^{EC}} + \frac{P^{EO}}{K_d^{EO}} \tag{S56}$$

$$\frac{1}{K_d^{ens(Fab\text{-}stabilized\ states)}} = \frac{P^{BC \cdot Fab}}{K_d^{BC}} + \frac{P^{EC \cdot Fab}}{K_d^{EC}} + \frac{P^{EO \cdot Fab}}{K_d^{EO}} \tag{S57}$$

Eq. S56–S57 relate the measurables $K_d^{ens(Basal)}$ and $K_d^{ens(Fab\text{-}stabilized\ states)}$ to the probabilities (populations) and intrinsic ligand-binding affinities of each state in the basal ensemble, forming the basis for their calculation described in the next section. The rest of this section will examine specific cases of $K_d^{ens(Fab\text{-}stabilized\ states)}$ determined with different Fabs (Eq. S55).

Three-state ensembles

Open-stabilizing Fabs (O Fab) selectively stabilize the EO state, i.e., [BC·Fab] = 0 and [EC·Fab] = 0, hence $P^{BC \cdot Fab} = 0$, $P^{EC \cdot Fab} = 0$ and $P^{EO \cdot Fab} = 1$ (from Eq. S50–S52). Therefore, $K_d^{ens(Fab\text{-}stabilized\ states)} = K_d^{EO}$ (from Eq. S57), and Eq. S55 becomes:

$$K_d^{EO} = \frac{P^{\alpha_5\beta_1 \cdot Fab} K_d^{ens(Basal)} K_d^{app(O\ Fab)}}{K_d^{ens(Basal)} - \left(1 - P^{\alpha_5\beta_1 \cdot Fab}\right) K_d^{app(O\ Fab)}} \approx K_d^{app(O\ Fab)} \tag{S58}$$

Fig. S2 shows that $P^{\alpha_5\beta_1 \cdot Fab}$ was > 95% for all open-stabilizing Fab used here at concentrations shown in Table S1, and that $K_d^{app(O\ Fab)}$ is indistinguishable from K_d^{EO}; i.e., the difference between these quantities is smaller than the experimental error in K_d^{EO}.

Extension-stabilizing Fabs (E Fab) stabilize both EC and EO states, i.e., [BC·Fab] = 0. Denoting $K_d^{ens(Fab\text{-}stabilized\ states)}$ as $K_d^{ens(EC+EO)}$, Eq. S55 becomes:

$$K_d^{ens(EC+EO)} = \frac{P^{\alpha_5\beta_1 \cdot Fab} K_d^{ens(Basal)} K_d^{app(E\ Fab)}}{K_d^{ens(Basal)} - \left(1 - P^{\alpha_5\beta_1 \cdot Fab}\right) K_d^{app(E\ Fab)}} \approx K_d^{app(E\ Fab)} \tag{S59}$$

As in the case of open-stabilizing Fabs, Fig. S2 shows that $P^{\alpha_5\beta_1 \cdot Fab}$ was > 95% for all extension-stabilizing Fab used here at concentrations shown in Table S1, and that $K_d^{app(E\ Fab)}$ is indistinguishable from $K_d^{ens(EC+EO)}$; i.e., the difference between these quantities is smaller than the experimental error in $K_d^{ens(EC+EO)}$.

From Eq. S50–S52, S32 and S35–S36:

$$P^{BC \cdot Fab} = 0 \tag{S60}$$

$$P^{\mathrm{EC\cdot Fab}} = \frac{[\mathrm{EC\cdot Fab}]}{[\mathrm{EC\cdot Fab}] + [\mathrm{EO\cdot Fab}]} = \frac{[\mathrm{EC}]}{[\mathrm{EC}] + [\mathrm{EO}]} = \frac{P^{\mathrm{EC}}}{P^{\mathrm{EC}} + P^{\mathrm{EO}}} \tag{S61}$$

$$P^{\mathrm{EO\cdot Fab}} = \frac{[\mathrm{EO\cdot Fab}]}{[\mathrm{EC\cdot Fab}] + [\mathrm{EO\cdot Fab}]} = \frac{[\mathrm{EO}]}{[\mathrm{EC}] + [\mathrm{EO}]} = \frac{P^{\mathrm{EO}}}{P^{\mathrm{EC}} + P^{\mathrm{EO}}} \tag{S62}$$

Thus, from Eq. S57:

$$\frac{1}{K_{\mathrm{d}}^{\mathrm{ens(EC+EO)}}} = \frac{P^{\mathrm{EC}}}{P^{\mathrm{EC}} + P^{\mathrm{EO}}} \cdot \frac{1}{K_{\mathrm{d}}^{\mathrm{EC}}} + \frac{P^{\mathrm{EO}}}{P^{\mathrm{EC}} + P^{\mathrm{EO}}} \cdot \frac{1}{K_{\mathrm{d}}^{\mathrm{EO}}} \tag{S63}$$

Closure-stabilizing Fabs (C Fab) stabilize both BC and EC states, i.e., [EO·Fab] = 0. Denoting $K_{\mathrm{d}}^{\mathrm{ens(Fab\text{-}stabilized\ states)}}$ as $K_{\mathrm{d}}^{\mathrm{ens(BC+EC)}}$, Eq. S55 becomes:

$$K_{\mathrm{d}}^{\mathrm{ens(BC+EC)}} = \frac{P^{\alpha_5\beta_1\cdot\mathrm{Fab}} K_{\mathrm{d}}^{\mathrm{ens(Basal)}} K_{\mathrm{d}}^{\mathrm{app(C\ Fab)}}}{K_{\mathrm{d}}^{\mathrm{ens(Basal)}} - \left(1 - P^{\alpha_5\beta_1\cdot\mathrm{Fab}}\right) K_{\mathrm{d}}^{\mathrm{app(C\ Fab)}}} \tag{S64}$$

Fig. S2 shows that $P^{\alpha_5\beta_1\cdot\mathrm{Fab}}$ was around 99.8–99.9% for all closure-stabilizing Fab used here at concentrations shown in Table S1, and that $K_{\mathrm{d}}^{\mathrm{app(C\ Fab)}}$ is appreciably different from $K_{\mathrm{d}}^{\mathrm{ens(BC+EC)}}$. Therefore, $K_{\mathrm{d}}^{\mathrm{ens(BC+EC)}}$ was calculated from $P^{\alpha_5\beta_1\cdot\mathrm{Fab}}$, $K_{\mathrm{d}}^{\mathrm{ens(Basal)}}$ and $K_{\mathrm{d}}^{\mathrm{app(C\ Fab)}}$ using Eq. S64.

From Eq. S50–S52, S32 and S34–S35:

$$P^{\mathrm{BC\cdot Fab}} = \frac{[\mathrm{BC\cdot Fab}]}{[\mathrm{BC\cdot Fab}] + [\mathrm{EC\cdot Fab}]} = \frac{[\mathrm{BC}]}{[\mathrm{BC}] + [\mathrm{EC}]} = \frac{P^{\mathrm{BC}}}{P^{\mathrm{BC}} + P^{\mathrm{EC}}} \tag{S65}$$

$$P^{\mathrm{EC\cdot Fab}} = \frac{[\mathrm{EC\cdot Fab}]}{[\mathrm{BC\cdot Fab}] + [\mathrm{EC\cdot Fab}]} = \frac{[\mathrm{EC}]}{[\mathrm{BC}] + [\mathrm{EC}]} = \frac{P^{\mathrm{EC}}}{P^{\mathrm{BC}} + P^{\mathrm{EC}}} \tag{S66}$$

$$P^{\mathrm{EO\cdot Fab}} = 0 \tag{S67}$$

Thus, from Eq. S57:

$$\frac{1}{K_{\mathrm{d}}^{\mathrm{ens(BC+EC)}}} = \frac{P^{\mathrm{BC}}}{P^{\mathrm{BC}} + P^{\mathrm{EC}}} \cdot \frac{1}{K_{\mathrm{d}}^{\mathrm{BC}}} + \frac{P^{\mathrm{EC}}}{P^{\mathrm{BC}} + P^{\mathrm{EC}}} \cdot \frac{1}{K_{\mathrm{d}}^{\mathrm{EC}}} \tag{S68}$$

Extension-stabilizing Fab plus closure-stabilizing Fab (E+C Fabs) selectively stabilize the EC state, i.e., [BC·Fab] = 0 and [EO·Fab] = 0, hence $P^{\mathrm{BC\cdot Fab}} = 0$, $P^{\mathrm{EC\cdot Fab}} = 1$ and $P^{\mathrm{EO\cdot Fab}} = 0$ (from Eq. S50–S52). Therefore, $K_{\mathrm{d}}^{\mathrm{ens(Fab\text{-}stabilized\ states)}} = K_{\mathrm{d}}^{\mathrm{EC}}$ (from Eq. S57), and Eq. S55 becomes:

$$K_{\mathrm{d}}^{\mathrm{EC}} = \frac{P^{\alpha_5\beta_1\cdot\mathrm{Fab}} K_{\mathrm{d}}^{\mathrm{ens(Basal)}} K_{\mathrm{d}}^{\mathrm{app(E+C\ Fabs)}}}{K_{\mathrm{d}}^{\mathrm{ens(Basal)}} - \left(1 - P^{\alpha_5\beta_1\cdot\mathrm{Fab}}\right) K_{\mathrm{d}}^{\mathrm{app(E+C\ Fabs)}}} \tag{S69}$$

Two-state ensembles

For $\alpha_5\beta_1$ headpiece, the basal ensemble comprises only two overall conformational states: closed (C) and open (O). Similar analysis shows that the ensemble's ligand-binding affinity is (using dissociation constants):

$$\frac{1}{K_{\mathrm{d}}^{\mathrm{ens(Basal;\ HP)}}} = \frac{P^{\mathrm{C}}}{K_{\mathrm{d}}^{\mathrm{C}}} + \frac{P^{\mathrm{O}}}{K_{\mathrm{d}}^{\mathrm{O}}} \tag{S70}$$

where $K_{\mathrm{d}}^{\mathrm{C}}$, $K_{\mathrm{d}}^{\mathrm{O}}$, P^{C} and P^{O} are intrinsic ligand-binding affinities and probabilities (populations) of the closed and open headpiece conformations, respectively.

Open-stabilizing Fabs (O Fab) selectively stabilize the open headpiece. Therefore,

$K_d^{\text{ens(Fab-stabilized states)}} = K_d^{\text{O}}$ and

$$K_d^{\text{O}} = \frac{P^{\alpha_5\beta_1\cdot\text{Fab}} K_d^{\text{ens(Basal; HP)}} K_d^{\text{app(O Fab)}}}{K_d^{\text{ens(Basal; HP)}} - \left(1 - P^{\alpha_5\beta_1\cdot\text{Fab}}\right) K_d^{\text{app(O Fab)}}} \approx K_d^{\text{app(O Fab)}} \tag{S71}$$

$P^{\alpha_5\beta_1\cdot\text{Fab}}$ was 98.2% for the open-stabilizing Fab 12G10 used here at 2000 nM (Table S1), and Fig. S2 shows that $K_d^{\text{app(O Fab)}}$ is indistinguishable from K_d^{O}; i.e., the difference between these quantities is smaller than the experimental error in K_d^{O}.

Closure-stabilizing Fabs (C Fab) selectively stabilize the closed headpiece. Therefore, $K_d^{\text{ens(Fab-stabilized states)}} = K_d^{\text{C}}$ and

$$K_d^{\text{C}} = \frac{P^{\alpha_5\beta_1\cdot\text{Fab}} K_d^{\text{ens(Basal; HP)}} K_d^{\text{app(C Fab)}}}{K_d^{\text{ens(Basal; HP)}} - \left(1 - P^{\alpha_5\beta_1\cdot\text{Fab}}\right) K_d^{\text{app(C Fab)}}} \approx K_d^{\text{app(C Fab)}} \tag{S72}$$

Fig. S2 shows that $P^{\alpha_5\beta_1\cdot\text{Fab}}$ was > 99.8% for all closure-stabilizing Fab used here at concentrations shown in Table S1, and that $K_d^{\text{app(C Fab)}}$ is indistinguishable from K_d^{C}; i.e., the difference between these quantities is smaller than the experimental error in K_d^{C}.

Calculation of probability of each conformational state and Eqs. S73–S77

The experimentally determined ligand-binding affinities $K_d^{\text{ens(Basal)}}$ (measured in the absence of Fab), K_d^{EO} (Eq. S58), $K_d^{\text{ens(EC+EO)}}$ (Eq. S59) and K_d^{EC} (Eq. S69) relate to the probabilities P^{BC}, P^{EC} and P^{EO} through Eq. S56 and S63, which are repeated here for convenience:

$$\frac{1}{K_d^{\text{ens(Basal)}}} = \frac{P^{\text{BC}}}{K_d^{\text{BC}}} + \frac{P^{\text{EC}}}{K_d^{\text{EC}}} + \frac{P^{\text{EO}}}{K_d^{\text{EO}}} \tag{S56 revisited}$$

$$\frac{1}{K_d^{\text{ens(EC+EO)}}} = \frac{P^{\text{EC}}}{P^{\text{EC}} + P^{\text{EO}}} \cdot \frac{1}{K_d^{\text{EC}}} + \frac{P^{\text{EO}}}{P^{\text{EC}} + P^{\text{EO}}} \cdot \frac{1}{K_d^{\text{EO}}} \tag{S63 revisited}$$

In addition, the measured $K_d^{\text{ens(BC+EC)}}$ (Eq. S64) and K_d^{EC} are virtually indistinguishable, suggesting that $K_d^{\text{BC}} \simeq K_d^{\text{EC}} \simeq K_d^{\text{ens(BC+EC)}}$. Therefore, K_d^{BC} and K_d^{EC} are approximated by $K_d^{\text{ens(BC+EC)}}$. Since $K_d^{\text{ens(BC+EC)}}$ is much larger than $K_d^{\text{ens(Basal)}}$, $K_d^{\text{ens(EC+EO)}}$ and K_d^{EO}, this approximation and the relatively large uncertainty in $K_d^{\text{ens(BC+EC)}}$ have no impact on the calculated probabilities (Fig. S2).

Solve these equations for the probabilities, noting that $P^{\text{BC}} + P^{\text{EC}} + P^{\text{EO}} = 1$ (Eq. S37):

$$P^{\text{EO}} = \frac{K_d^{\text{EO}}\left(K_d^{\text{ens(BC+EC)}} - K_d^{\text{ens(Basal)}}\right)}{K_d^{\text{ens(Basal)}}\left(K_d^{\text{ens(BC+EC)}} - K_d^{\text{EO}}\right)} \tag{S73}$$

$$P^{\text{EC}} = \frac{K_d^{\text{ens(BC+EC)}}\left(K_d^{\text{ens(BC+EC)}} - K_d^{\text{ens(Basal)}}\right)\left(K_d^{\text{ens(EC+EO)}} - K_d^{\text{EO}}\right)}{K_d^{\text{ens(Basal)}}\left(K_d^{\text{ens(BC+EC)}} - K_d^{\text{EO}}\right)\left(K_d^{\text{ens(BC+EC)}} - K_d^{\text{ens(EC+EO)}}\right)} \tag{S74}$$

$$P^{\mathrm{BC}} = \frac{K_{\mathrm{d}}^{\mathrm{ens(BC+EC)}}\left(K_{\mathrm{d}}^{\mathrm{ens(Basal)}} - K_{\mathrm{d}}^{\mathrm{ens(EC+EO)}}\right)}{K_{\mathrm{d}}^{\mathrm{ens(Basal)}}\left(K_{\mathrm{d}}^{\mathrm{ens(BC+EC)}} - K_{\mathrm{d}}^{\mathrm{ens(EC+EO)}}\right)} \tag{S75}$$

Likewise, for $\alpha_5\beta_1$ headpiece

$$P^{\mathrm{O}} = \frac{K_{\mathrm{d}}^{\mathrm{O}}\left(K_{\mathrm{d}}^{\mathrm{C}} - K_{\mathrm{d}}^{\mathrm{ens(Basal;\ HP)}}\right)}{K_{\mathrm{d}}^{\mathrm{ens(Basal;\ HP)}}\left(K_{\mathrm{d}}^{\mathrm{C}} - K_{\mathrm{d}}^{\mathrm{O}}\right)} \tag{S76}$$

$$P^{\mathrm{C}} = \frac{K_{\mathrm{d}}^{\mathrm{C}}\left(K_{\mathrm{d}}^{\mathrm{ens(Basal;\ HP)}} - K_{\mathrm{d}}^{\mathrm{O}}\right)}{K_{\mathrm{d}}^{\mathrm{ens(Basal;\ HP)}}\left(K_{\mathrm{d}}^{\mathrm{C}} - K_{\mathrm{d}}^{\mathrm{O}}\right)} \tag{S77}$$

Calculation of free energy of each conformational state and Eqs. S78–S84

Using EO as the reference state ($\Delta G^{\mathrm{EO}} = 0$), the relative free energies of the BC and EC states, ΔG^{BC} and ΔG^{EC}, are related to the probabilities (populations) P^{BC}, P^{EC} and P^{EO} through the Boltzmann distribution (as also shown in Fig. 1C):

$$P^{\mathrm{BC}} = \frac{1}{Q}\exp\left(-\frac{\Delta G^{\mathrm{BC}}}{RT}\right) \tag{S78}$$

$$P^{\mathrm{EC}} = \frac{1}{Q}\exp\left(-\frac{\Delta G^{\mathrm{EC}}}{RT}\right) \tag{S79}$$

$$P^{\mathrm{EO}} = \frac{1}{Q} \tag{S80}$$

$$Q = 1 + \exp\left(-\frac{\Delta G^{\mathrm{BC}}}{RT}\right) + \exp\left(-\frac{\Delta G^{\mathrm{EC}}}{RT}\right) \tag{S81}$$

where Q is known as the partition function.

Solve Eq. S78–S81 for ΔG^{BC} and ΔG^{EC}, and substituting P^{BC}, P^{EC} and P^{EO} with Eq. S73–S75:

$$\Delta G^{\mathrm{BC}} = -RT\ln\left(\frac{P^{\mathrm{BC}}}{P^{\mathrm{EO}}}\right) = -RT\ln\frac{K_{\mathrm{d}}^{\mathrm{ens(BC+EC)}}\left(K_{\mathrm{d}}^{\mathrm{ens(BC+EC)}} - K_{\mathrm{d}}^{\mathrm{EO}}\right)\left(K_{\mathrm{d}}^{\mathrm{ens(Basal)}} - K_{\mathrm{d}}^{\mathrm{ens(EC+EO)}}\right)}{K_{\mathrm{d}}^{\mathrm{EO}}\left(K_{\mathrm{d}}^{\mathrm{ens(BC+EC)}} - K_{\mathrm{d}}^{\mathrm{ens(Basal)}}\right)\left(K_{\mathrm{d}}^{\mathrm{ens(BC+EC)}} - K_{\mathrm{d}}^{\mathrm{ens(EC+EO)}}\right)} \tag{S82}$$

$$\Delta G^{\mathrm{EC}} = -RT\ln\left(\frac{P^{\mathrm{EC}}}{P^{\mathrm{EO}}}\right) = -RT\ln\frac{K_{\mathrm{d}}^{\mathrm{ens(BC+EC)}}\left(K_{\mathrm{d}}^{\mathrm{ens(EC+EO)}} - K_{\mathrm{d}}^{\mathrm{EO}}\right)}{K_{\mathrm{d}}^{\mathrm{EO}}\left(K_{\mathrm{d}}^{\mathrm{ens(BC+EC)}} - K_{\mathrm{d}}^{\mathrm{ens(EC+EO)}}\right)} \tag{S83}$$

Likewise, for $\alpha_5\beta_1$ headpiece, using O as the reference state ($\Delta G^{\mathrm{O}} = 0$)

$$\Delta G^{\mathrm{C}} = -RT\ln\left(\frac{P^{\mathrm{C}}}{P^{\mathrm{O}}}\right) = -RT\ln\frac{K_{\mathrm{d}}^{\mathrm{C}}\left(K_{\mathrm{d}}^{\mathrm{ens(Basal;\ HP)}} - K_{\mathrm{d}}^{\mathrm{O}}\right)}{K_{\mathrm{d}}^{\mathrm{O}}\left(K_{\mathrm{d}}^{\mathrm{C}} - K_{\mathrm{d}}^{\mathrm{ens(Basal;\ HP)}}\right)} \tag{S84}$$

Calculation of free energies associated with conformational changes and Eqs. S85–S94

$\alpha_5\beta_1$ activation is associated with ectodomain extension and headpiece opening. These two types of conformational changes are not necessarily separate, independent steps; nor must they occur in a predefined order, allowing the conformational change among the three integrin states to be defined as from one state to another, or as interchange between one state and two other states. Indeed, we have previously described scenarios for different orders of steps [7], and movies show how headpiece opening may either follow or precede extension (Supplemental Movies EV1–EV3). States such as bent-open may also be possible (Movie EV3); however, since these states have never been visualized by electron microscopy or small-angle X-ray scattering, their populations must be small, and thus the presence of their populations in the numerator or denominator of K_{conf} (defined below) has little effect on K_{conf} values.

Extension from BC to EC defines $K_{\mathrm{conf}}^{\mathrm{E}}$ and its associated free energy $\Delta G_{\mathrm{conf}}^{\mathrm{E}}$:

$$\mathrm{BC} \rightleftharpoons \mathrm{EC} \qquad K_{\mathrm{conf}}^{\mathrm{E}} = \frac{[\mathrm{EC}]}{[\mathrm{BC}]} = \frac{P^{\mathrm{EC}}}{P^{\mathrm{BC}}} \tag{S85}$$

$$\Delta G_{\mathrm{conf}}^{\mathrm{E}} = -RT \ln K_{\mathrm{conf}}^{\mathrm{E}} = -RT \ln \frac{P^{\mathrm{EC}}}{P^{\mathrm{BC}}} = -RT \ln \frac{\left(K_{\mathrm{d}}^{\mathrm{ens(BC+EC)}} - K_{\mathrm{d}}^{\mathrm{ens(Basal)}}\right)\left(K_{\mathrm{d}}^{\mathrm{ens(EC+EO)}} - K_{\mathrm{d}}^{\mathrm{EO}}\right)}{\left(K_{\mathrm{d}}^{\mathrm{ens(BC+EC)}} - K_{\mathrm{d}}^{\mathrm{EO}}\right)\left(K_{\mathrm{d}}^{\mathrm{ens(Basal)}} - K_{\mathrm{d}}^{\mathrm{ens(EC+EO)}}\right)} \tag{S86}$$

Opening from EC to EO defines $K_{\mathrm{conf}}^{\mathrm{O}}$ and its associated free energy $\Delta G_{\mathrm{conf}}^{\mathrm{O}}$:

$$\mathrm{EC} \rightleftharpoons \mathrm{EO} \qquad K_{\mathrm{conf}}^{\mathrm{O}} = \frac{[\mathrm{EO}]}{[\mathrm{EC}]} = \frac{P^{\mathrm{EO}}}{P^{\mathrm{EC}}} \tag{S87}$$

$$\Delta G_{\mathrm{conf}}^{\mathrm{O}} = -RT \ln K_{\mathrm{conf}}^{\mathrm{O}} = -RT \ln \frac{P^{\mathrm{EO}}}{P^{\mathrm{EC}}} = -RT \ln \frac{K_{\mathrm{d}}^{\mathrm{ens(BC+EC)}} - K_{\mathrm{d}}^{\mathrm{EO}}}{K_{\mathrm{d}}^{\mathrm{EO}}\left(K_{\mathrm{d}}^{\mathrm{ens(BC+EC)}} - K_{\mathrm{d}}^{\mathrm{ens(EC+EO)}}\right)} \tag{S88}$$

With these definitions, $\Delta G_{\mathrm{conf}}^{\mathrm{E}}$ and $\Delta G_{\mathrm{conf}}^{\mathrm{O}}$ sum to $-\Delta G^{\mathrm{BC}}$.

Alternatively, extension from BC to either EC or EO defines $K_{\mathrm{conf}}^{\mathrm{E\,tot}}$ and its associated free energy $\Delta G_{\mathrm{conf}}^{\mathrm{E\,tot}}$:

$$\left.\begin{array}{l}\mathrm{BC} \rightleftharpoons \mathrm{EC} \\ \mathrm{BC} \rightleftharpoons \mathrm{EO}\end{array}\right\} \quad K_{\mathrm{conf}}^{\mathrm{E\,tot}} = \frac{[\mathrm{EC}] + [\mathrm{EO}]}{[\mathrm{BC}]} = \frac{P^{\mathrm{EC}} + P^{\mathrm{EO}}}{P^{\mathrm{BC}}} \tag{S89}$$

$$\Delta G_{\mathrm{conf}}^{\mathrm{E\,tot}} = -RT \ln K_{\mathrm{conf}}^{\mathrm{E\,tot}} = -RT \ln \frac{P^{\mathrm{EC}} + P^{\mathrm{EO}}}{P^{\mathrm{BC}}} = -RT \ln \frac{K_{\mathrm{d}}^{\mathrm{ens(BC+EC)}}\left(K_{\mathrm{d}}^{\mathrm{ens(Basal)}} - K_{\mathrm{d}}^{\mathrm{ens(EC+EO)}}\right)}{K_{\mathrm{d}}^{\mathrm{ens(EC+EO)}}\left(K_{\mathrm{d}}^{\mathrm{ens(BC+EC)}} - K_{\mathrm{d}}^{\mathrm{ens(Basal)}}\right)} \tag{S90}$$

Extension-and-opening from either BC or EC to EO defines $K_{\mathrm{conf}}^{\mathrm{Activation}}$ and its associated free energy $\Delta G_{\mathrm{conf}}^{\mathrm{Activation}}$:

$$\left.\begin{array}{l}\mathrm{BC} \rightleftharpoons \mathrm{EO} \\ \mathrm{EC} \rightleftharpoons \mathrm{EO}\end{array}\right\} \quad K_{\mathrm{conf}}^{\mathrm{Activation}} = \frac{[\mathrm{EO}]}{[\mathrm{BC}] + [\mathrm{EC}]} = \frac{P^{\mathrm{EO}}}{P^{\mathrm{BC}} + P^{\mathrm{EC}}} \tag{S91}$$

$$\Delta G_{\mathrm{conf}}^{\mathrm{Activation}} = -RT \ln K_{\mathrm{conf}}^{\mathrm{Activation}} = -RT \ln \frac{P^{\mathrm{EO}}}{P^{\mathrm{BC}} + P^{\mathrm{EC}}} = -RT \ln \frac{K_{\mathrm{d}}^{\mathrm{EO}}\left(K_{\mathrm{d}}^{\mathrm{ens(BC+EC)}} - K_{\mathrm{d}}^{\mathrm{ens(Basal)}}\right)}{K_{\mathrm{d}}^{\mathrm{ens(BC+EC)}}\left(K_{\mathrm{d}}^{\mathrm{ens(Basal)}} - K_{\mathrm{d}}^{\mathrm{EO}}\right)} \tag{S92}$$

For $\alpha_5\beta_1$ headpiece, the only conformational change is opening from C to O, which defines $K_{\mathrm{conf}}^{\mathrm{O;\,HP}}$ and its associated free energy $\Delta G_{\mathrm{conf}}^{\mathrm{O;\,HP}}$:

$$\mathrm{C} \rightleftharpoons \mathrm{O} \qquad K_{\mathrm{conf}}^{\mathrm{O;\,HP}} = \frac{[\mathrm{O}]}{[\mathrm{C}]} = \frac{P^{\mathrm{O}}}{P^{\mathrm{C}}} \tag{S93}$$

$$\Delta G_{\mathrm{conf}}^{\mathrm{O;\,HP}} = -RT \ln K_{\mathrm{conf}}^{\mathrm{O;\,HP}} = -RT \ln \frac{P^{\mathrm{O}}}{P^{\mathrm{C}}} = -RT \ln \frac{K_{\mathrm{d}}^{\mathrm{O}}\left(K_{\mathrm{d}}^{\mathrm{C}} - K_{\mathrm{d}}^{\mathrm{ens(Basal;\,HP)}}\right)}{K_{\mathrm{d}}^{\mathrm{C}}\left(K_{\mathrm{d}}^{\mathrm{ens(Basal;\,HP)}} - K_{\mathrm{d}}^{\mathrm{O}}\right)} \tag{S94}$$

Supplemental References

1. Huang, C., and Springer, T.A. (1997). Folding of the β-propeller domain of the integrin α_L subunit is independent of the I domain and dependent on the β_2 subunit. Proc Natl Acad Sci U S A *94*, 3162–3167.

2. Su, Y., Xia, W., Li, J., Walz, T., Humphries, M.J., Vestweber, D., Cabañas, C., Lu, C., and Springer, T.A. (2016). Relating conformation to function in integrin $\alpha_5\beta_1$. Proc Natl Acad Sci U S A. 3. Freyer, M.W., and Lewis, E.A. (2008). Isothermal titration calorimetry: experimental design, data analysis, and probing macromolecule/ligand binding and kinetic interactions. Methods Cell Biol *84*, 79–113.

4. Babul, J., and Stellwagen, E. (1969). Measurement of protein concentration with interferences optics. Anal Biochem *28*, 216–221.

5. Mohri, H. (1996). Fibronectin and integrins interactions. J Investig Med *44*, 429-441.

6. Rossi, A.M., and Taylor, C.W. (2011). Analysis of protein-ligand interactions by fluorescence polarization. Nat Protoc *6*, 365–387.

7. Takagi, J., Petre, B.M., Walz, T. and Springer, T.A. (2002). Global conformational rearrangements in integrin extracellular domains in outside-in and inside-out signaling. Cell *110*, 5, 599–611.

Supplemental Figures

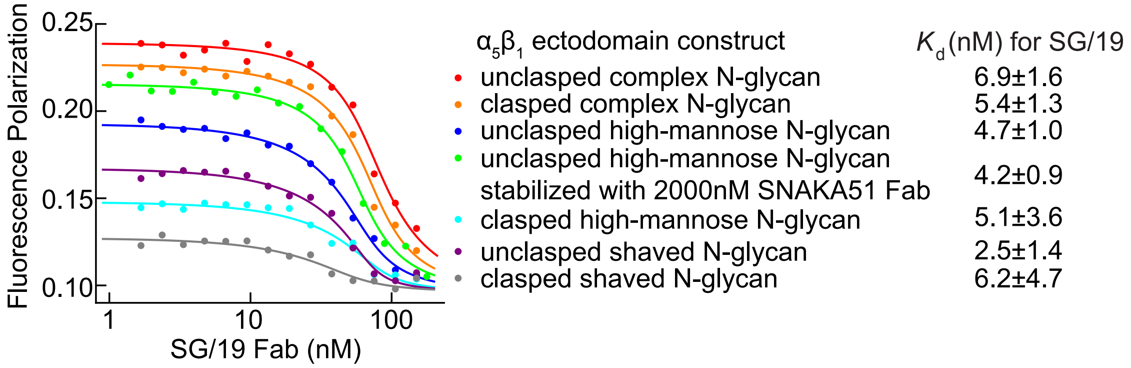

Fig. S1. Affinities of SG/19 Fab for $\alpha_5\beta_1$ ectodomain preparations.
Binding of closure-stabilizing Fab SG/19 to $\alpha_5\beta_1$ (100 nM) influenced binding of FITC-cRGD (5 nM) to $\alpha_5\beta_1$ as monitored by FP. K_d values were obtained from fitting the FP data to Eq. S11. Errors are fitting errors from triplicates.

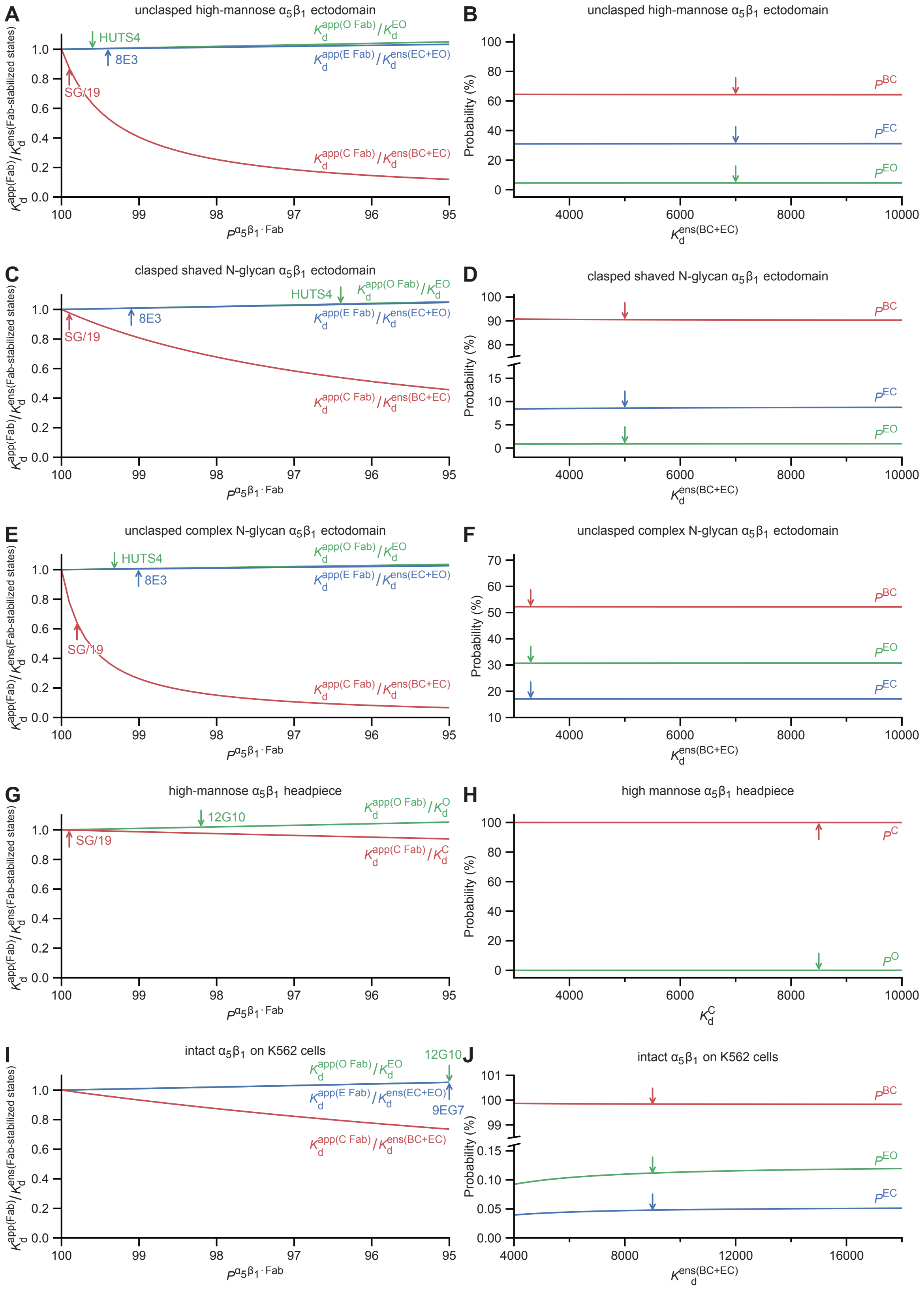

Fig. S2. Accuracy in determination of the thermodynamic parameters in $\alpha_5\beta_1$ ensembles.
(A, C, E, G and I) The ratio of apparent ligand-binding affinity in the presence of Fabs ($K_d^{app(Fab)}$) to that

of the ensemble comprising Fab-stabilized states only ($K_d^{\text{ens(Fab-stabilized states)}}$, Eq. S55) plotted against the fraction of Fab-bound $\alpha_5\beta_1$ ($P^{\alpha_5\beta_1\cdot\text{Fab}}$). The plots were made with experimental values of $K_d^{\text{ens(Basal)}}$ and $K_d^{\text{ens(Fab-stabilized states)}}$, except $K_d^{\text{ens(BC+EC)}}$ for intact $\alpha_5\beta_1$ on K562 cells is estimated from K_d^{EO} using the same fold-difference as found with $\text{Fn3}_{9\text{–}10}$ for the $\alpha_5\beta_1$ ectodomain (Fig. 4D). Arrows show $P^{\alpha_5\beta_1\cdot\text{Fab}}$ values under the experimental conditions used with the indicated Fabs (Table S1). (B, D, F, H and J) Lack of sensitivity of the probability of each conformational state on the ensemble affinity of closed states ($K_d^{\text{ens(BC+EC)}}$). Plots were made from Eq. S73–S75, using experimental values of K_d^{EO}, $K_d^{\text{ens(EC+EO)}}$ and $K_d^{\text{ens(Basal)}}$. Arrows show experimental values of $K_d^{\text{ens(BC+EC)}}$ (or K_d^{C} for the headpiece). (A and B) Unclasped high-mannose N-glycan $\alpha_5\beta_1$ ectodomain. (C and D) Clasped shaved N-glycan $\alpha_5\beta_1$ ectodomain. (E and F) Unclasped complex N-glycan $\alpha_5\beta_1$ ectodomain. (G and H) High-mannose $\alpha_5\beta_1$ headpiece. (I and J) Intact $\alpha_5\beta_1$ on K562 cells. Although curves are not shown for all $\alpha_5\beta_1$ ectodomain, headpiece, and semi-truncated constructs, their curves are identical or intermediate between the examples shown here. Thus, the headpiece and semi-truncated construct have identical curves, and require the highest Fab concentrations for opening. Among ectodomains, the clasped shaved and the unclasped complex N-glycan ectodomains require the most and least energy to activate ($\Delta G_{\text{conf}}^{\text{Activation}}$), respectively.

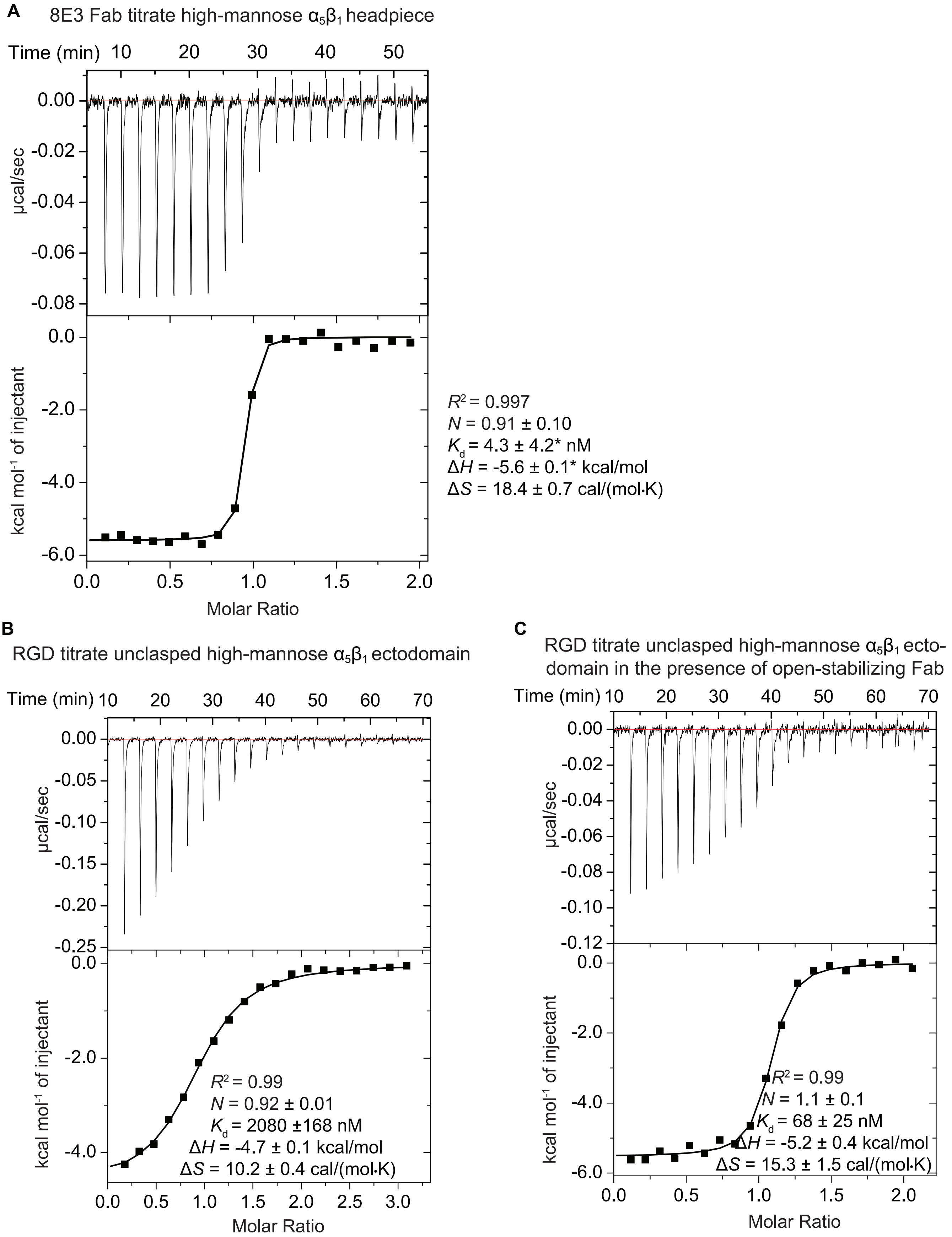
A
8E3 Fab titrate high-mannose α5β1 headpiece
Time (min) 10 20 30 40 50
0.00
-0.02
-0.04
-0.06
-0.08
µcal/sec
0.0
-2.0
-4.0
-6.0
kcal mol-1 of injectant
0.0 0.5 1.0 1.5 2.0
Molar Ratio
R2 = 0.997
N = 0.91 ± 0.10
Kd = 4.3 ± 4.2* nM
ΔH = -5.6 ± 0.1* kcal/mol
ΔS = 18.4 ± 0.7 cal/(mol·K)
B
RGD titrate unclasped high-mannose α5β1 ectodomain
Time (min) 10 20 30 40 50 60 70
0.00
-0.05
-0.10
-0.15
-0.20
-0.25
µcal/sec
0.0
-2.0
-4.0
kcal mol-1 of injectant
R2 = 0.99
N = 0.92 ± 0.01
Kd = 2080 ±168 nM
ΔH = -4.7 ± 0.1 kcal/mol
ΔS = 10.2 ± 0.4 cal/(mol·K)
0.0 0.5 1.0 1.5 2.0 2.5 3.0
Molar Ratio
C
RGD titrate unclasped high-mannose α5β1 ecto-
domain in the presence of open-stabilizing Fab
Time (min) 10 20 30 40 50 60 70
0.00
-0.02
-0.04
-0.06
-0.08
-0.10
-0.12
µcal/sec
0.0
-2.0
-4.0
-6.0
kcal mol-1 of injectant
R2 = 0.99
N = 1.1 ± 0.1
Kd = 68 ± 25 nM
ΔH = -5.2 ± 0.4 kcal/mol
ΔS = 15.3 ± 1.5 cal/(mol·K)
0.0 0.5 1.0 1.5 2.0
Molar Ratio

Fig. S3. Affinity of 8E3 Fab and RGD peptide for $\alpha_5\beta_1$ preparations by ITC.

(A) 10 μM $\alpha_5\beta_1$ titrated with 100 μM 8E3 Fab. (B) 25 μM $\alpha_5\beta_1$ titrated with 375 μM RGD peptide. (C) 10 μM $\alpha_5\beta_1$ titrated with 100 μM RGD peptide in the presence of 20 μM open-stabilizing Fab HUTS4. Data were fit to the one-site binding model (Freyer and Lewis, 2008) in OriginPro 7. Errors with "*" are s.d. from three independent measurements. Binding entropy ΔS was calculated from $\Delta G = RT \ln K_\mathrm{d} = \Delta H - T\Delta S$ and errors were propagated. Other errors without "*" are fitting errors.

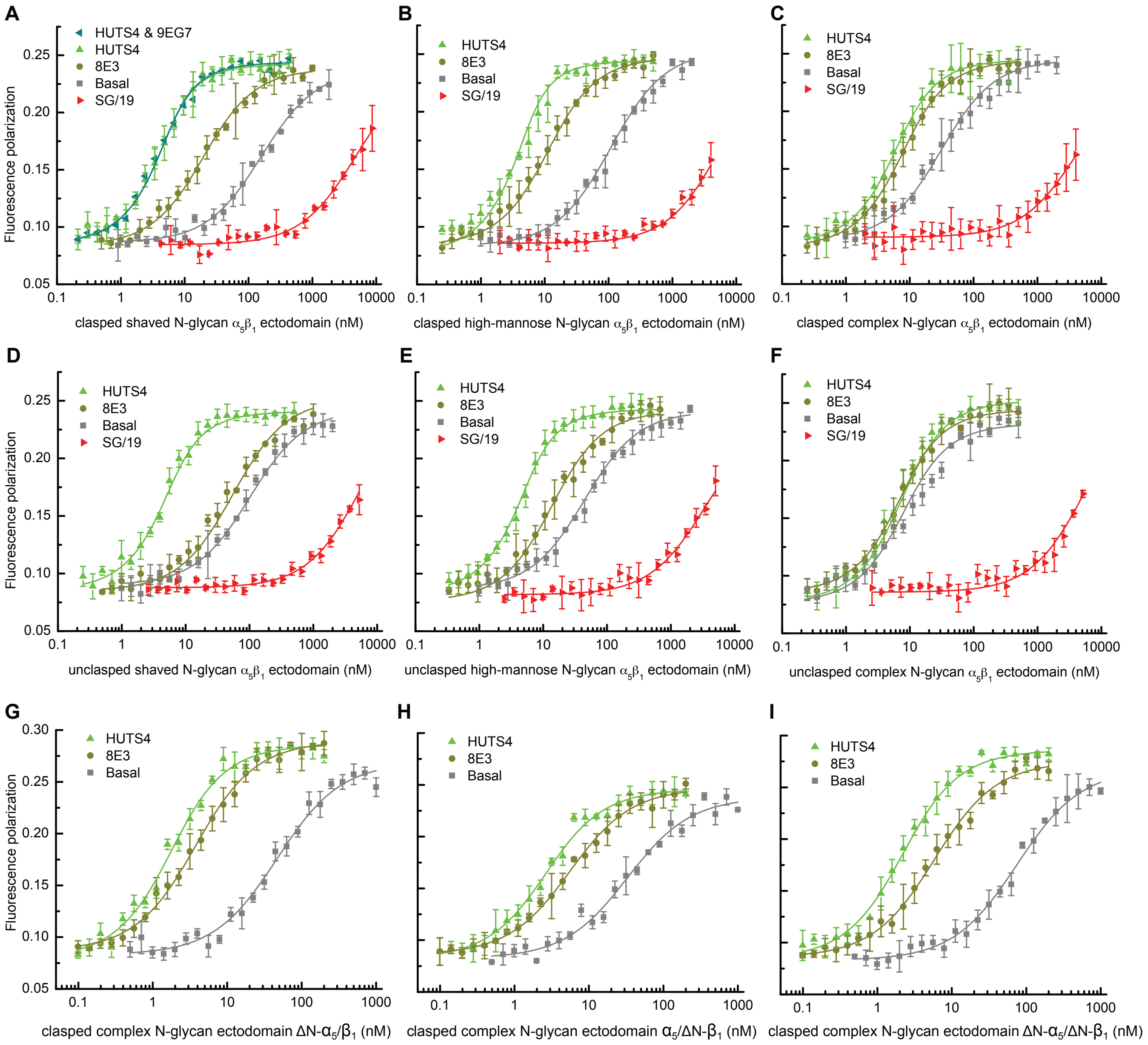

Fig. S4. Intrinsic and ensemble affinities of $\alpha_5\beta_1$ preparations for cRGD.
Clasped (A–C) and unclasped (D–F) $\alpha_5\beta_1$ ectodomain with shaved (A and D), high-mannose (B and E) and complex (C and F) N-glycans. (G–I) Clasped complex N-glycan $\alpha_5\beta_1$ ectodomain glycosylation site mutants ΔN-α_5/β_1 (G), α_5/ΔN-β_1 (H) and ΔN-α_5/ΔN-β_1 (I). Binding was measured in the absence of Fabs, or in the presence of 5 μM open-stabilizing Fab HUTS4, 2 μM extension-stabilizing Fab 8E3, or 10 μM closure-stabilizing Fab SG/19. Data were fit to Eq. S16. Error bars in the plots are s.d. of triplicates.

A

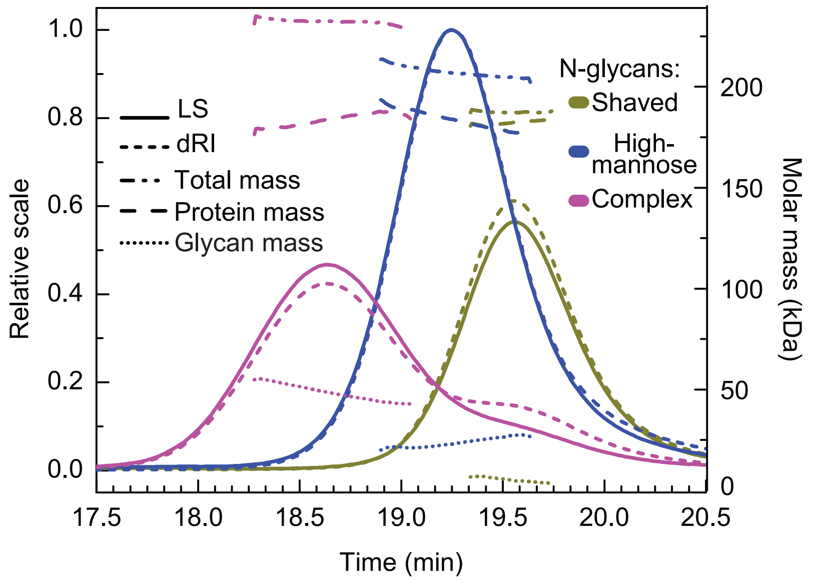

B Unclasped $\alpha_5\beta_1$ ectodomain
(protein molar mass from sequence: 184.7kDa)

	Complex N-glycan	High-mannose N-glycan	Shaved N-glycan
Total molar mass<br>Fitting error	232.2 kDa<br>(±0.236%)	207.2 kDa<br>(±0.245%)	187.5 kDa<br>(±0.196%)
Protein molar mass<br>Fitting error	183.6KDa<br>(±0.235%)	183.4kDa<br>(±0.246%)	182.2 kDa<br>(±0.196%)
Glycan molar mass<br>Fitting error	48.6 kDa<br>(±1.199%)	23.5kDa<br>(±2.105%)	5.0 kDa<br>(±8.283%)
Protein	79%	89%	97%
Glycan	21%	11%	3%

Fig. S5. Protein/carbohydrate composition of unclasped $\alpha_5\beta_1$ ectodomain with shaved, high-mannose and complex N-glycans.

(A) Gel filtration profile and molar mass analysis. Light scattering (LS) intensity and differential refractive index (dRI) were normalized to their maximum values. (B) Calculated protein, glycan, and total molar masses according to (A) using the protein conjugate model. Errors are fitting errors.

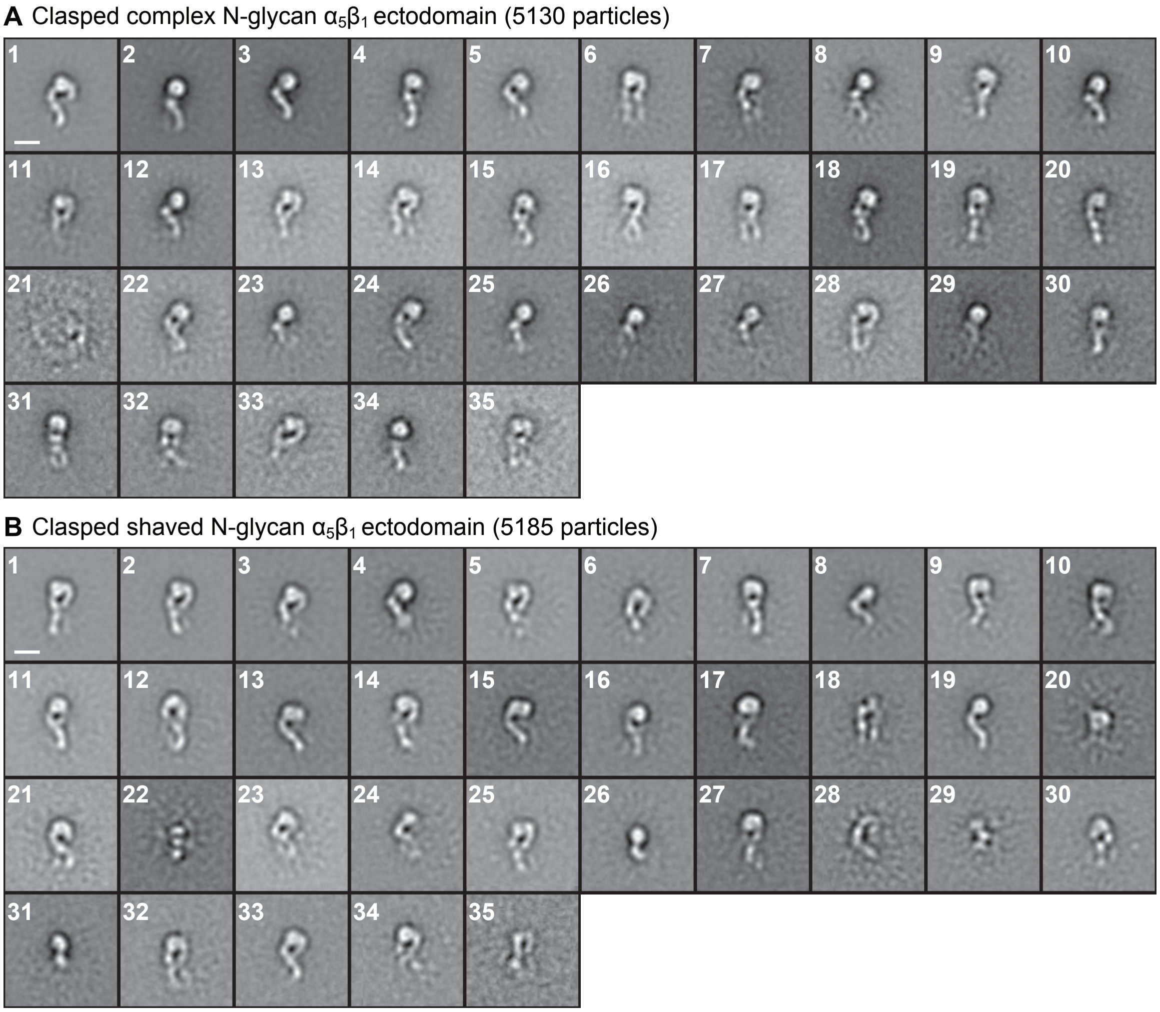

Fig. S6. EM of clasped $\alpha_5\beta_1$ ectodomains.
All class averages of clasped $\alpha_5\beta_1$ ectodomain with complex (A) and shaved (B) N-glycans. Scale bar is 10 nm.

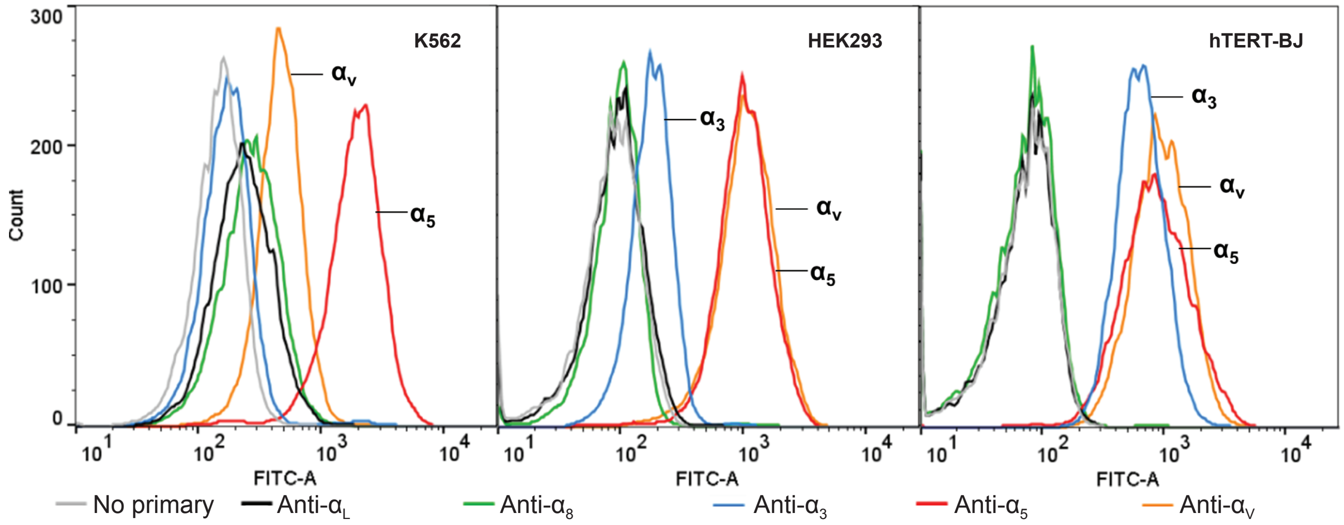

Fig. S7. Immuno-fluorescent staining of integrin α subunits on K562, HEK293 and hTERT-BJ cells analyzed by flow cytometry.

Cells were stained with 2.5 μg/mL primary antibody, followed by 2 μg/mL FITC-conjugated secondary antibody. Anti-α_L serves as negative control.

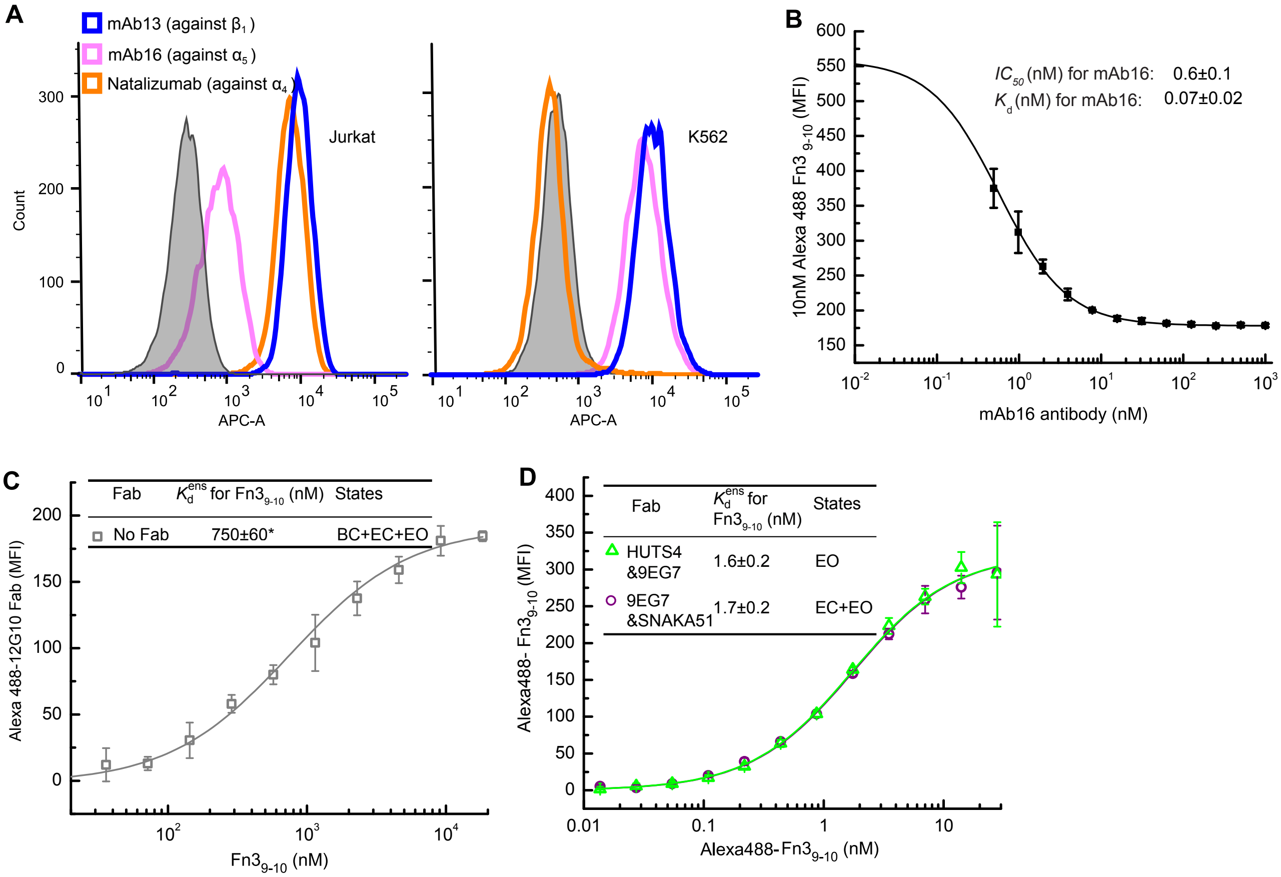

Fig. S8. Conformational equilibria and intrinsic affinity of intact $\alpha_5\beta_1$ on Jurkat cells.

(A) Immuno-fluorescent staining of integrin α_5, α_4 and β_1 subunits with Alexa647 labeled detection antibodies on Jurkat and K562 cells analyzed by flow cytometry. Anti-mouse IgG serves as negative control. (B) Affinities of mAb16, α_5-specific blocking antibody, for $\alpha_5\beta_1$. Binding of mAb16 to $\alpha_5\beta_1$ influenced binding of Alexa488-Fn_{9-10} (10 nM) to $\alpha_5\beta_1$ on K562 cells under saturating open-stabilizing Fab 9EG7 and HUTS4 as monitored by FACS. IC_{50} value was fitted to Eq. S2, errors are fitting errors from triplicates. K_d values were obtained by $K_d = \dfrac{IC_{50}}{K_d^{EO:Fn_{9-10}} + 1}$, where $K_d^{EO:Fn_{9-10}} = 1.4$ nM as determined in Fig.7B. Errors in K_d are propagated from errors in IC_{50} and $K_d^{EO:Fn_{9-10}}$. (C)Affinity of $\alpha_5\beta_1$ on Jurkat cells for Fn_{9-10} by enhancement of 0.4 nM Alexa488-12G10 Fab binding. (D) Affinity of $\alpha_5\beta_1$ on Jurkat cells for Alexa488-Fn_{9-10} in presence of indicated Fabs.

Table S1. Fab EC_{50} or K_d values, concentrations used in ligand-binding affinity measurements ($[\text{Fab}]_{tot}$), and probabilities of Fab-bound $\alpha_5\beta_1$ states ($P^{\alpha_5\beta_1\cdot\text{Fab}}$).

	Fab<br>Stabilized states	HUTS4<br>EO	12G10<br>EO	8E3<br>EC+EO	9EG7<br>EC+EO	N29<br>EC+EO	SNAKA51<br>EC+EO	SG/19<br>BC+EC	mAb13<br>BC+EC
high-mannose headpiece	EC_{50} (nM)	2800±600	28±3	4.3±4.2[1]				< 4.7[2]	< 2.3[2]
	$[\text{Fab}]_{tot}$ (nM)	unused	2000	20000		epitope not present		20000	20000
	$P^{\alpha_5\beta_1\cdot\text{Fab}}$ (%)		98.2	99.8				> 99.8	> 99.9
clasped complex N-glycan ectodomain	EC_{50} (nM)	34±4		15±3				5.4±1.3[3]	
	$[\text{Fab}]_{tot}$ (nM)	5000	unused	2000	unused	unused	unused	10000	unused
	$P^{\alpha_5\beta_1\cdot\text{Fab}}$ (%)	99.3		99.0				99.9	
unclasped complex N-glycan ectodomain	EC_{50} (nM)	< 34[4]		< 15[4]				6.9±1.6[3]	
	$[\text{Fab}]_{tot}$ (nM)	5000	unused	2000	unused	unused	unused	10000	unused
	$P^{\alpha_5\beta_1\cdot\text{Fab}}$ (%)	> 99.3		> 99.0				99.8	
clasped high-mannose ectodomain	EC_{50} (nM)	80±6		15±3				5.1±3.6[3]	
	$[\text{Fab}]_{tot}$ (nM)	5000	unused	2000	unused	unused	unused	10000	unused
	$P^{\alpha_5\beta_1\cdot\text{Fab}}$ (%)	98.3		99.0				99.9	
unclasped high-mannose ectodomain	EC_{50} (nM)	20±3	1.1±0.2	9±4	3.1±1.4	5.0±2.4	8.1±3.1	4.7±1.0[3]	2.3±1.0
	$[\text{Fab}]_{tot}$ (nM)	5000	1000	2000	2000	2000	2000	10000	15000
	$P^{\alpha_5\beta_1\cdot\text{Fab}}$ (%)	99.6	99.8	99.4	99.8	99.7	99.5	99.9	99.9
clasped shaved N-glycan ectodomain	EC_{50} (nM)	167±23		14±5				6.2±4.7[3]	
	$[\text{Fab}]_{tot}$ (nM)	5000	unused	2000	unused	unused	unused	10000	unused
	$P^{\alpha_5\beta_1\cdot\text{Fab}}$ (%)	96.4		99.1				99.9	
unclasped shaved N-glycan ectodomain	EC_{50} (nM)	49±6		6±5				2.5±1.4[3]	
	$[\text{Fab}]_{tot}$ (nM)	5000	unused	2000	unused	unused	unused	10000	unused
	$P^{\alpha_5\beta_1\cdot\text{Fab}}$ (%)	98.9		99.6				99.9	
intact $\alpha_5\beta_1$ on K562 cells	EC_{50} (nM)	2900±300	107±5		690±30				
	$[\text{Fab}]_{tot}$ (nM)	unused	2000	unused	13000	unused	unused	unused	unused
	$P^{\alpha_5\beta_1\cdot\text{Fab}}$ (%)		95.0		95.0				

	Fabs<br>Stabilized states	HUTS4+9EG7<br>EO	$Fn3_{9-10}$+9EG7<br>EO	9EG7+SNAKA51<br>EC+EO	mAb13+9EG7<br>EC	SG/19+SNAKA51<br>EC
unclasped high-mannose ectodomain	EC_{50} (nM)	< 20			1.8±0.6[5]	3.0±0.7[5]
	$[\text{Fab}]_{tot}$ (nM)	5000+2000	unused	unused	15000+10000	15000+10000
	$P^{\alpha_5\beta_1\cdot\text{Fab}}$ (%)	> 99.6			99.9	99.9
intact $\alpha_5\beta_1$ on K562 cells	EC_{50} (nM)	21±2[6]	2.1±0.1[7]	< 690±30[8]		
	$[\text{Fab}/\text{Fn3}_{9-10}]_{tot}$ (nM)	2000+6000	2000+13000	13000+2000	unused	unused
	$P^{\alpha_5\beta_1\cdot\text{Fab}}$ (%)	99.0	99.9	> 95.0		

[1] K_d value determined by ITC (Fig. S3A).

[2] K_d and EC_{50} values are shown as lower than those determined for the unclasped high-mannose ectodomain; they must be so based on Eq. S33, because the ~100-fold lower basal affinity of the high-mannose headpiece compared to the unclasped high-mannose ectodomain (Fig. 3) demonstrates a higher proportion of the closed conformation in the headpiece ensemble than in the unclasped high-mannose ectodomain ensemble.

[3] K_d value determined for SG/19 in the presence of 100 nM $\alpha_5\beta_1$ ectodomain (Fig. S1).

[4] EC_{50} values are shown as lower than those determined for the clasped complex N-glycan ectodomain; they must be so based on Eq. S33, because the ~2-fold higher basal affinity of the unclasped complex N-glycan ectodomain compared to the clasped complex N-glycan ectodomain (Fig. 5B) demonstrates a higher proportion of the EO conformation in the unclasped complex N-glycan ectodomain ensemble than in the clasped complex N-glycan ectodomain ensemble.

[5] K_d value for mAb13 or SG/19, measured in the presence of 100 nM unclasped high-mannose $\alpha_5\beta_1$ ectodomain and 2000 nM 9EG7 or SNAKA51 (representative data in Fig. S1).

[6] Measured for HUTS4 in the presence of 6000 nM 9EG7 (Fig. 7A).

[7] K_d value is determined for $Fn3_{9-10}$ in the presence of 13000 nM 9EG7 on K562 cells (Fig. 7B).

[8] EC_{50} value for 9EG7 in the presence of its synergic Fab SNAKA51 (Su et al., 2016), inferred from 9EG7 Fab's EC_{50} value under basal condition.
