# Supplementary material for: Conformational equilibria and intrinsic affinities define integrin activation
Source: EMBO J. 2017 Jan 25;36(5):629–45. doi: 10.15252/embj.201695803 (PMC5331762; doi:10.15252/embj.201695803)
Supplement: Supplementary file 2 — Movie EV1 [file EMBJ-36-629-s002.zip › Movie_EV1_Legend.docx]

**Movie EV1**. Conformational changes involved in integrin activation, scenario 1.

Conformational changes begin with separation of the lower legs, followed by ectodomain extension, and lastly headpiece opening.
