# Supplementary material for: Conformational equilibria and intrinsic affinities define integrin activation
Source: EMBO J. 2017 Jan 25;36(5):629–45. doi: 10.15252/embj.201695803 (PMC5331762; doi:10.15252/embj.201695803)
Supplement: Supplementary file 3 — Movie EV2 [file EMBJ-36-629-s003.zip › Movie_EV2_Legend.docx]

**Movie EV2**. Conformational changes involved in integrin activation, scenario 2.

Conformational changes begin with ectodomain extension, followed by headpiece opening, and lastly separation of the lower legs.
